# Supplementary material for: Different forms of superspreading lead to different outcomes: Heterogeneity in infectiousness and contact behavior relevant for the case of SARS-CoV-2
Source: PLoS Comput Biol. 2022 Aug 22;18(8):e1009980. doi: 10.1371/journal.pcbi.1009980 (PMC9436127; doi:10.1371/journal.pcbi.1009980)
Supplement: S1 Text — (PDF) [file pcbi.1009980.s001.pdf]

S1 Text. Population and parameters.

Elise J. Kuylen[1,2*], Andrea Torneri[1], Lander Willem[1], Pieter J. K. Libin[2,3,4], Steven Abrams[2,5], Pietro Coletti[2], Nicolas Franco[2,6], Frederik Verelst[1], Philippe Beutels[1,7], Jori Liesenborgs[8], Niel Hens[1,2]

1 Centre for Health Economic Research and Modeling Infectious Diseases, University of Antwerp, Antwerp, Belgium
2 Data Science Institute, I-BioStat, Hasselt University, Hasselt, Belgium
3 Artificial Intelligence Lab, Vrije Universiteit Brussel, Brussels, Belgium
4 Rega Institute for Medical Research, Clinical and Epidemiological Virology, University of Leuven, Leuven, Belgium
5 Global Health Institute, University of Antwerp, Antwerp, Belgium
6 Namur Institute for Complex Systems, Department of Mathematics, University of Namur, Namur, Belgium
7 School of Public Health and Community Medicine, The University of New South Wales, Sydney, NSW, Australia
8 Expertise Centre for Digital Media, Hasselt University - transnational University Limburg, Hasselt, Belgium

* elise.kuylen@uantwerpen.be

Population

We used a synthetic population of 11 million individuals, representing the total population of Belgium. The population is constructed once before all simulations are conducted. No births, deaths, immigration or emigration occur over the course of the simulations – making our population a closed one.

Household sizes and age constitutions were based on census data collected in Belgium in 2011 [1]. The geographic location of households is based on census data collected in 2001 [2]. Individuals in the population were assigned to schools and workplaces based on their geographic location, and school attendance and commuting data [2, 3],. Furthermore, each individual was assigned to two communities – to represent other contacts made during respectively the week and the weekend. These communities consist of, on average, 500 individuals, and are also based on geographic location.

Social contact rates

Pre-pandemic contact rates were based on a social contact study conducted in Belgium in 2010 and 2011 [4]. We assumed households to be fully connected (i.e. the contact probability between two household members is always 0.999) [5].

Symptomatic individuals reduce their contacts at the workplace and at school by 90%, and have 75% less contacts in their community contact pools [6].

Contact reductions during lockdown periods, and periods of partial release were estimated based on social contact data collected for Belgium in the CoMix study [7]. To obtain contact rates during the lockdown period, we used results from surveys conducted from April to mid-May 2020, while we used results from surveys conducted from mid-May to August 2020 to obtain contact rates during the period of partial release.

Contact rates collected in the CoMix study were subdivided into different categories. We used contact rates from the ‘work’ category to estimate contact reductions in workplace contact pools, and contact rates from the ‘transport’, ‘leisure’, and ‘other’ categories to estimate contact reductions in the community social contact pools.

To estimate the contact reductions, we aggregated the contact rates during the lockdown period and the partial release phase over all ages, and compared these rates to the pre-pandemic contact rates as obtained from the social contact study conducted in 2010–2011 [4].

Natural history of COVID-19

The natural history of COVID-19 was implemented in accordance with an earlier study conducted with STRIDE [8]. Following infection, individuals experience a latent period followed by either a pre-symptomatic and a symptomatic infectious period, or a fully asymptomatic infectious period.

The length of the incubation period (from the moment of infection to the start of the symptomatic/asymptomatic period) is drawn from a discretized log-normal distribution with logmean=1.43 and logsd=0.66 [9, 10]. The length of the pre-symptomatic infectious period follows a Gamma distribution, with shape=20.52, rate=1.59 and shift=12.27, truncated at -1 and divided by the cumulative distribution function [9]. The total length of the infectious period is drawn from a normal distribution with mean=6 and sd=1 [9, 11]. Finally, individuals that experience symptoms are symptomatic for 7 days, before they eventually recover [9].

Asymptomatic cases and individuals during the pre-symptomatic phase are assumed to be half as infectious as individuals experiencing symptoms [10].

The probability that an infected individual becomes symptomatic is age-dependent, and is calculated in the same way as for a previous study by Willem et al. [8, 10, 12].

Furthermore, we assumed that children are as susceptible as adults.

Finally, we assumed that when individuals recovered, they acquired immunity against infection for the duration of the simulation.

Other relevant parameters

We chose February 17th 2020 as the start date for all simulations, and used holidays and school holidays as applicable in Belgium during the simulated period (2020–2021).

For further information about the model, and the manner in which it was adapted to model the transmission of SARS-CoV-2 we refer to previous work by Kuylen et al. (2017) and Willem et al. (2021) [8, 13].

References

1. Census 2011; 2011. Accessible from: `https://census2011.fgov.be/`.

2. Census 2001; 2001. Accessible from: `https://statbel.fgov.be/nl/over-statbel/wat-doen-we/volkstellingen-census/census-publicaties`.

3. Commission E. EUROSTAT: Your Key to European Statistics;. Accessible from: `https://ec.europa.eu/eurostat/data/`.

4. Van Hoang T, Coletti P, Kifle YW, Van Kerckhove K, Vercruysse S, Willem L, et al. Close contact infection dynamics over time: insights from a second

large-scale social contact survey in Flanders, Belgium, in 2010–2011. BMC infectious diseases. 2021;21(1):1–15.

5. Goeyvaerts N, Santermans E, Potter G, Torneri A, Van Kerckhove K, Willem L, et al. Household members do not contact each other at random: implications for infectious disease modelling. Proceedings of the Royal Society B. 2018;285(1893):20182201.

6. Van Kerckhove K, Hens N, Edmunds JW, Eames KTD. The impact of illness on social networks: Implications for transmission and control of influenza. American Journal of Epidemiology. 2013;178(11):1655–1662.

7. Coletti P, Wambua J, Gimma A, Willem L, Vercruysse S, Vanhoutte B, et al. CoMix: comparing mixing patterns in the Belgian population during and after lockdown. Scientific Reports. 2020;10(1):21885.

8. Willem L, Abrams S, Libin PJ, Coletti P, Kuylen E, Petrof O, et al. The impact of contact tracing and household bubbles on deconfinement strategies for COVID-19. Nature Communications. 2021;12(1):1–9.

9. He X, Lau EHY, Wu P, Xilong D, Wang J, Hao X, et al. Temporal dynamics in viral shedding and transmissibility of COVID-19. Nature Medicine. 2020;26.

10. Li Q, Guan X, Wu P, Wang X, Zhou L, Tong Y, et al. Early transmission dynamics in Wuhan, China, of novel coronavirus–infected pneumonia. New England journal of medicine. 2020;.

11. Lourenco J, Paton R, Ghafari M, Kraemer M, Thompson C, Simmonds P, et al. Fundamental principles of epidemic spread highlight the immediate need for large-scale serological surveys to assess the stage of the SARS-CoV-2 epidemic. MedRxiv. 2020;.

12. Wu JT, Leung K, Bushman M, Kishore N, Niehus R, de Salazar PM, et al. Estimating clinical severity of COVID-19 from the transmission dynamics in Wuhan, China. Nature medicine. 2020;26(4):506–510.

13. Kuylen E, Stijven S, Broeckhove J, Willem L. Social contact patterns in an individual-based simulator for the transmission of infectious diseases (stride). Procedia Computer Science. 2017;108:2438–2442.
