# Supplementary material for: Different forms of superspreading lead to different outcomes: Heterogeneity in infectiousness and contact behavior relevant for the case of SARS-CoV-2
Source: PLoS Comput Biol. 2022 Aug 22;18(8):e1009980. doi: 10.1371/journal.pcbi.1009980 (PMC9436127; doi:10.1371/journal.pcbi.1009980)
Supplement: S2 Text — (PDF) [file pcbi.1009980.s002.pdf]

S2 Text. Verification.

Elise J. Kuylen[1,2*], Andrea Torneri[1], Lander Willem[1], Pieter J. K. Libin[2,3,4], Steven Abrams[2,5], Pietro Coletti[2], Nicolas Franco[2,6], Frederik Verelst[1], Philippe Beutels[1,7], Jori Liesenborgs[8], Niel Hens[1,2]

1 Centre for Health Economic Research and Modeling Infectious Diseases, University of Antwerp, Antwerp, Belgium
2 Data Science Institute, I-BioStat, Hasselt University, Hasselt, Belgium
3 Artificial Intelligence Lab, Vrije Universiteit Brussel, Brussels, Belgium
4 Rega Institute for Medical Research, Clinical and Epidemiological Virology, University of Leuven, Leuven, Belgium
5 Global Health Institute, University of Antwerp, Antwerp, Belgium
6 Namur Institute for Complex Systems, Department of Mathematics, University of Namur, Namur, Belgium
7 School of Public Health and Community Medicine, The University of New South Wales, Sydney, NSW, Australia
8 Expertise Centre for Digital Media, Hasselt University - transnational University Limburg, Hasselt, Belgium

* elise.kuylen@uantwerpen.be

Distribution of secondary cases and P_{80}

We verified that the mean R_0 remained stable over the different scenarios. To estimate R_0, we looked at the mean of the secondary cases made by each index case in the simulations for the scenarios without interventions. In the violin plots in Fig A, the distribution and mean of the number of secondary cases caused per index case is shown. Variations in in the mean between the different scenarios are small, and are likely caused by stochasticity, as no upward or downward trend can be observed.

We observe that, when increasing contact-related heterogeneity, the distribution of the estimated R_0 is less concentrated towards zero, and the maximum number of secondary cases is lower than it is when infectiousness-related heterogeneity is increased. As such, overdispersion seems to be less pronounced for the same values of α_c compared to α_i.

Once we were assured that the mean R_0 remained stable, we also verified that the variation in transmission changed accordingly when we increased either infectiousness-related or contact-related heterogeneity. To do this, we calculated P_{80} (see Fig B), or the minimal proportion of infected individuals that is responsible for 80% of transmissions.

We observe that indeed, as we increase either infectiousness-related (Fig B1) or contact-related (Fig B2) heterogeneity, P_{80} decreases, indicating that a smaller proportion of infected individuals is responsible for 80% of new cases. However, the effect is stronger when infectiousness-related heterogeneity is increased. When α_i is decreased from 10 to 0.2, P_{80} decreases from 0.30 to 0.12, whereas P_{80} decreases from 0.31 to 0.23 when α_c is decreased from 10 to 0.2. As such, we need a very high level of contact-related heterogeneity, or a certain level of infectiousness-related heterogeneity to obtain the range for P_{80} (between 0.1 and 0.2) that was estimated for SARS-CoV-2 [1].

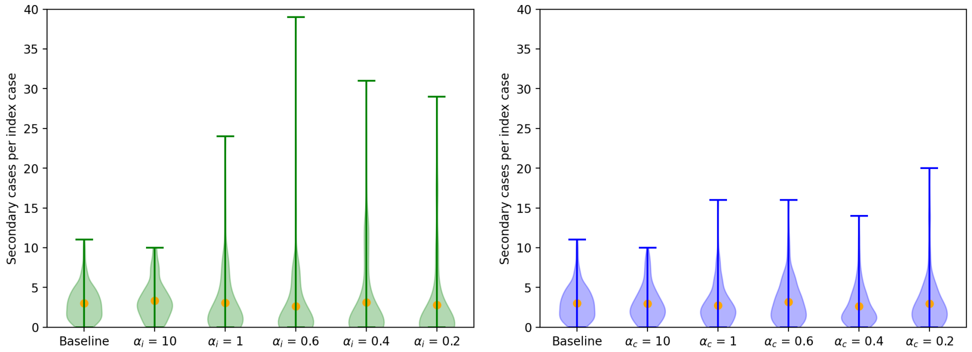

(1) Varying α_i for the Truncated Gamma distribution considered for the individual transmission probability.

(2) Varying α_c for the Gamma distribution considered the individual contact factor.

Fig A. Violin plots for the number of secondary cases per index case for the different scenarios regarding infectiousness related heterogeneity (in green, panel 1) and contact-related heterogeneity (in blue, panel 2). The orange dots represent the means of the simulated values.

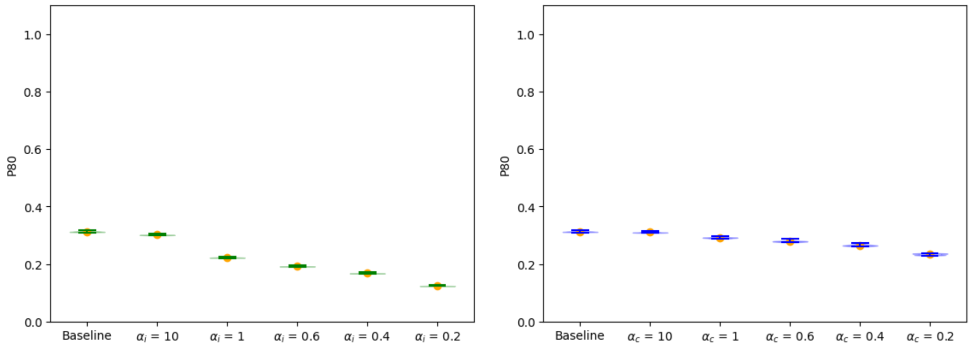

(1) Varying α_i for the Truncated Gamma distribution considered for the individual transmission probability.

(2) Varying α_c for the Gamma distribution considered for the individual contact factor.

Fig B. Violin plots for P_{80} over the different scenarios regarding infectiousness-related (in green, panel 1) and contact-related (in blue, panel 2) heterogeneity. Runs which led to extinction (i.e. in which less than 20 cases occurred) were excluded.

Finally, note that in the baseline case, P_{80} is only 0.31, meaning that there already is a certain level of heterogeneity in transmission. This can be explained by the fact that in STRIDE, social mixing is never completely homogeneous, as contact rates are dependent on the ages of individuals and the locations in which contacts take place.

Theoretical description

Theoretical description

We constructed a theoretical description to estimate the mean and variance of the number of secondary cases per infected individual. Let i be a member of the (finite) population in Stride. Individual i is assigned to different contact pools, such as a household or a workplace, in which they interact with other members of the population. We indicate the set of individuals that belong to a contact pool k, together with individual i, as L_k^i. Then the collection of contact pools of which i is a member, i.e. $\left\{L_k^i\right\}_k$, characterizes all individuals in the population that i can contact.

In such a contact pool, individuals have a probability to contact other members. This probability depends on the age of the individuals, and the type of contact pool. Individuals who are members of the same contact pool have at most one contact per simulation day, according to a Bernouilli distribution. When a contact is realized between an infected and a susceptible individual, this contact can then result in an infection event with a defined probability – and as such this also follows a Bernouilli distribution.

We now consider i an individual in a completely susceptible population, and j and individual that shares a contact pool $\hat{k}$ with i, i.e. $j \in L_{\hat{k}}^i$. Assuming that i starts their infectious period on day $t = 0$, and that they are infectious for d days. In a single time-step, the probability that i has an effective contact with j can then be written as:

$$\begin{aligned}
&\mathbb{P}(\text{“i has an effective contact with j”}) \\
=&\mathbb{P}(\text{“i has a contact with j”} \cap \text{“the contact between i and j is effective”}) \\
=&\mathbb{P}(\text{“the contact between i and j is effective”} \mid \text{“i has a contact with j”}) \\
&\times \mathbb{P}(\text{“i has a contact with j”}) \\
=&q_{i,j} c_{i,j}
\end{aligned}$$

With $q_{i,j}$ the probability that a contact between i and j is effective, and $c_{i,j}$ is the probability that the individuals have a contact during the given time-step. According to this description, the number of effective contacts between i and j, $X^{i,j}$, can be described by a Binomial distribution, $X^{i,j} \sim Bin(d, q_{i,j}c_{i,j})$, under the assumption that effective contact events are realized independently during the infectious period. When this is the case, we can write the probability that i has at least one effective contact with j as:

$$\begin{aligned}
&\mathbb{P}(\text{“i has at least one effective contact with j”}) \\
&= \mathbb{P}(X^{i,j} \geq 1) \\
&= 1 - \mathbb{P}(X^{i,j} < 1) \\
&= 1 - \mathbb{P}(X^{i,j} = 0) \\
&= 1 - (1 - q_{i,j}c_{i,j})^d
\end{aligned}$$

Since we assume that the entire population, except for the index case i, is susceptible, the first effective contact between i and j will result in an infection event.

Thus, the probability that i infects j, $Y^{i,j}$, can be described by a Bernoulli random variable, $Y^{i,j} \sim Be(1-(1-q_{i,j}c_{i,j})^d)$. This description can be extended to account for all contacts that i can possibly have in a given contact pool. The number of secondary cases that i eventually makes in $\hat{k}$, $Y_{\hat{k}}$ is then given by:

$$Y_{\hat{k}} = \sum_{j \in L^i_{\hat{k}}, j \neq i} Y^{i,j}$$

Assuming that each $Y^{i,j}$ is independent from the others, $Y^{\hat{k}}$ follows a Poisson Binomial Distribution, with mean, μ, and variance σ^2, given by:

$$\mu_{\hat{k}} = \sum_{j \in L^i_{\hat{k}}, j \neq i} 1-(1-q_{i,j}c_{i,j})^d = |L^i_{\hat{k}}| - \sum_{j \in L^i_{\hat{k}}, j \neq i} (1-q_{i,j}c_{i,j})^d$$
$$\sigma^2_{\hat{k}} = \sum_{j \in L^i_{\hat{k}}, j \neq i} (1-q_{i,j}c_{i,j})^d(1-(1-q_{i,j}c_{i,j})^d)$$

Furthermore, the total number of secondary cases that i can possibly generate within a susceptible population, Y^i, can be written as:

$$Y^i = \sum_{k \in K^i} \left(\sum_{j \in L^i_{\hat{k}}, j \neq i} Y^{i,j} \right)$$

Wit K^i the set of contact pools to which i belongs. Same as above, Y^i, follows a Poisson Binomial distribution, with mean μ_i and variance σ^2_i, given by:

$$\mu_i = \sum_{k \in K^i} \sum_{j \in L^i_{\hat{k}}, j \neq i} 1-(1-q_{i,j}c_{i,j})^d$$
$$\sigma^2_i = \sum_{k \in K^i} \sum_{j \in L^i_{\hat{k}}, j \neq i} (1-q_{i,j}c_{i,j})^d(1-(1-q_{i,j}c_{i,j})^d)$$

Heterogeneity in disease transmission was modeled on the one hand by treating the transmission probability as a random variable, and the contact probability on the other hand.

If the individual transmission probability is modeled as a random variable, we can adapt the theoretical description as follows. We assume here that q is determined only by the infectious individual and is not dependent on the (susceptible) individual that this first individual has a contact with. As such, we will henceforth refer to q as q_i. Let q_i be a continuous random variable with support on $(0,1]$ and let $f_q(x)$ be its probability density function. Then the mean number of secondary cases individual i makes during their infectious period can be expressed as:

$$\mu_i = \mathbb{E}\left[\mathbb{E}[Y^i|q_i]\right] = \int_0^\infty \Big(\sum_{k \in K^i} \sum_{j \in L^i_{\hat{k}}, j \neq i} 1-(1-xc_{i,j})^d f_q(x)dx \Big)$$

Assuming that q follows a Truncated Gamma distribution defined on $(0,1]$ we obtain:

$$\bar{\mu}_i = \mathbb{E}\left[\mathbb{E}[Y^i|q_i]\right] = \int_0^1 \Big(\sum_{k \in K^i} \sum_{j \in L^i_{\hat{k}}, j \neq i} 1-(1-xc_{i,j})^d \frac{g(x)}{G(1)-G(0)} dx \Big)$$

Where $g(x), G(x)$ are respectively the density and the cumulative distribution function of the considered Gamma distribution.
The variance:

$$\sigma_i^2 = Var(Y^i) = \mathbb{E}[Var(Y^i|q)] + Var(\mathbb{E}[Y^i|q]) =$$
$$= \int_0^1 \Big(\sum_{k \in K^i} \sum_{j \in L_k^i, j \neq i} (1 - xc_{i,j})^d (1 - (1 - xc_{i,j})^d) \frac{g(x)}{G(1) - G(0)} dx \Big) +$$
$$+ \int_0^1 \Big(\sum_{k \in K^i} \sum_{j \in L_k^i, j \neq i} 1 - (1 - xc_{i,j})^d - \bar{\mu}_i \Big)^2 \frac{g(x)}{G(1) - G(0)} dx$$

Conversely, when the individual contact factor is treated as a random variable, this can be expressed as:

$$\mu_i = \mathbb{E}\left[\mathbb{E}[Y^i|cf_{i,j}]\right] = \sum_{k \in K^i} \sum_{j \in L_k^i, j \neq i} \int_0^\infty \Big(1 - (1 - q_{i,j}(y_{i,j}))^d f_{y_{i,j}}(y_{i,j}) dy_{i,j} \Big)$$

where $y_{i,j} = xc_{i,j}$ is the random variable that describes the contact rate between i and j.

Simulation

We compared the estimations calculated using the theoretical description to results obtained by simulation. In order to keep the theoretical description tractable, we introduced some simplification to the simulations that we ran to compare to the theoretical description.

First, we used a constant infectious period of 7 days. Secondly, symptomatic cases did not remain in their household, but kept having the same contact rates as before infection. Finally, we treated each simulation day as a weekday – so no weekends or holidays were modeled in these simulations.

We performed these simulations for different individuals: an adult (aged 46 years), a child (aged 5 years) and an elderly person (aged 75 years). We tested the following mean transmission probabilities: 0.025, 0.050, 0.075 and 0.10. As for our other experiments, we tested several values for α_i (10, 1, 0.6, 0.4, 0.2) as well as for α_c (10, 1, 0.6, 0.4, 0.2). We ran each simulation for 40 days, after which the index case should have recovered.

For each scenario, we ran 200 simulations.

Results

We compared theoretical estimates with simulation results for the mean and variance of the number of secondary cases per index case for an adult (see Fig C–F), a child (see Fig G–J) and an elderly person (see Fig K–N).

In all cases, we found that means estimated using the theoretical description closely matched the means of the simulation results. For the variance, the theoretically estimated quantities did not exactly match the simulated ones, but in both results a similar trend could be distinguished.

References

1. Kremer C, Torneri A, Boesmans S, Meuwissen H, Verdonschot S, Vanden Driessche K, et al. Quantifying superspreading for COVID-19 using Poisson mixture distributions. Scientific reports. 2021;11(1):1–11.

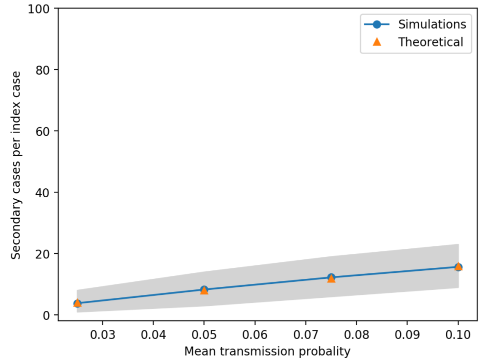

(1) Baseline

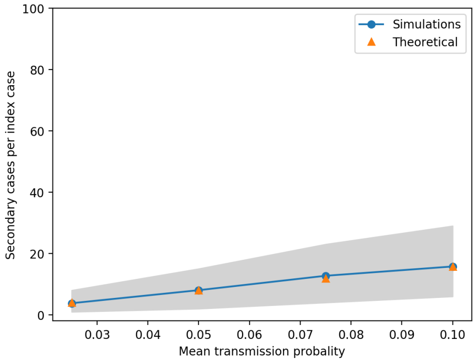

(2) $\alpha_i = 10$

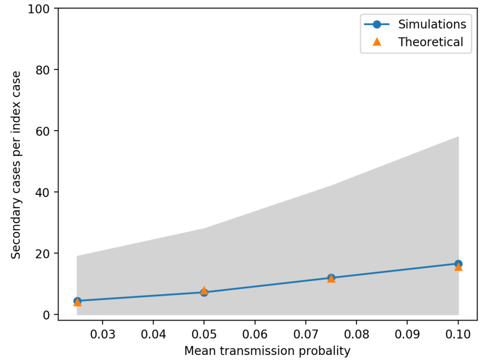

(3) $\alpha_i = 1$

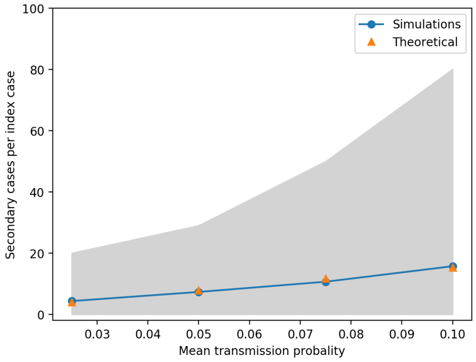

(4) $\alpha_i = 0.6$

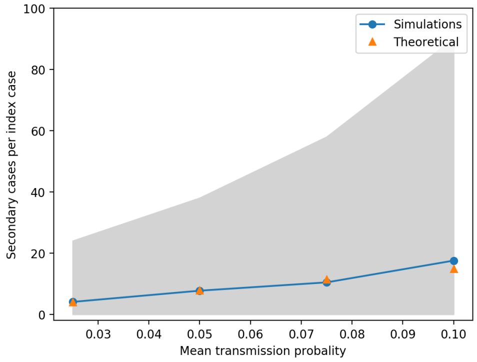

(5) $\alpha_i = 0.4$

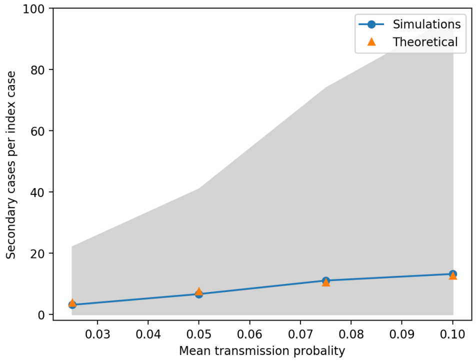

(6) $\alpha_i = 0.2$

Fig C. Comparison of the theoretical estimate for the mean individual reproduction number of an adult and the mean number of secondary cases per index case from simulations for the same individual for varying levels of infectiousness-related heterogeneity. Different values of α_i for the Truncated Gamma distribution considered for the individual transmission probability were tested.

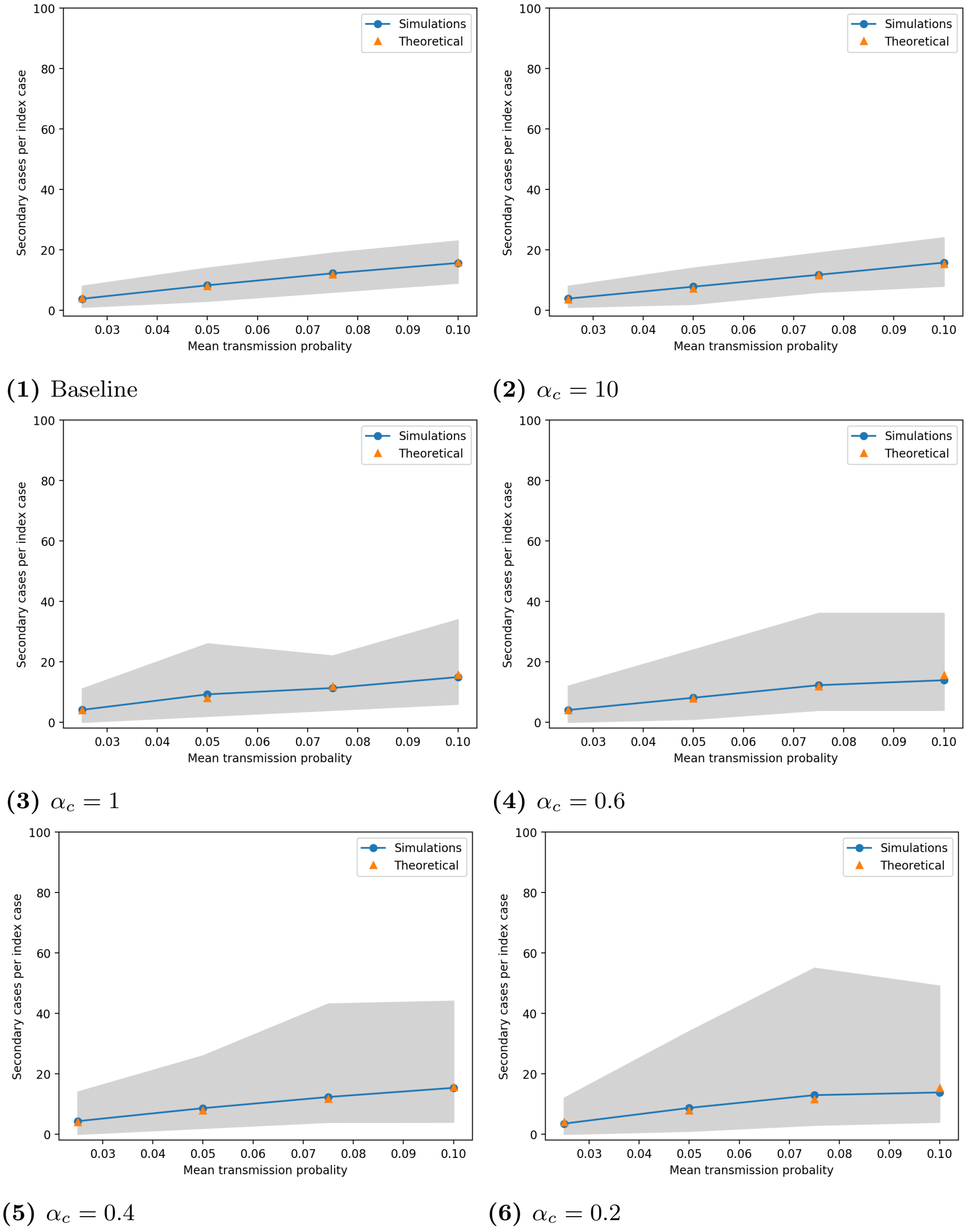

(1) Baseline **(2)** $\alpha_c = 10$

(3) $\alpha_c = 1$ **(4)** $\alpha_c = 0.6$

(5) $\alpha_c = 0.4$ **(6)** $\alpha_c = 0.2$

Fig D. Comparison of the theoretical estimate for the mean individual reproduction number of an adult and the mean number of secondary cases per index case from simulations for the same individual for varying levels of contact-related heterogeneity. Different values of α_c for the Gamma distribution considered for the individual contact factor were tested.

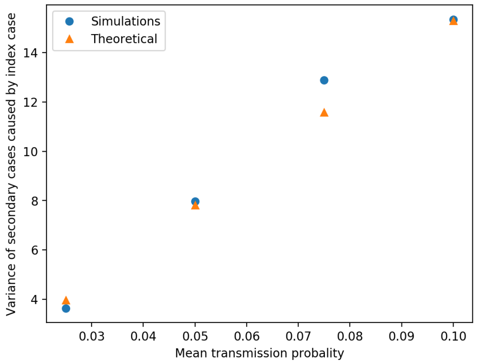

(1) Baseline

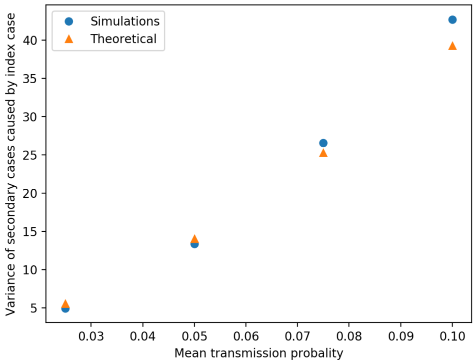

(2) $\alpha_i = 10$

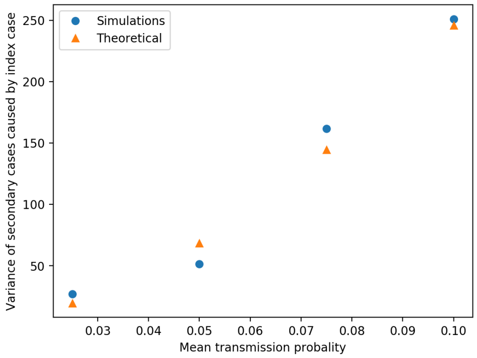

(3) $\alpha_i = 1$

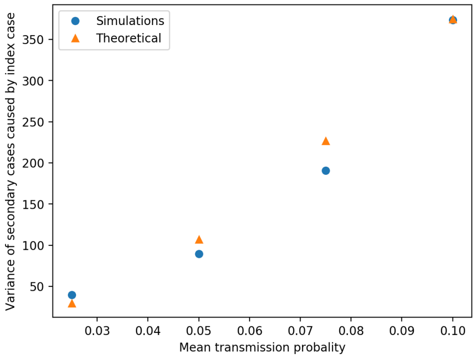

(4) $\alpha_i = 0.6$

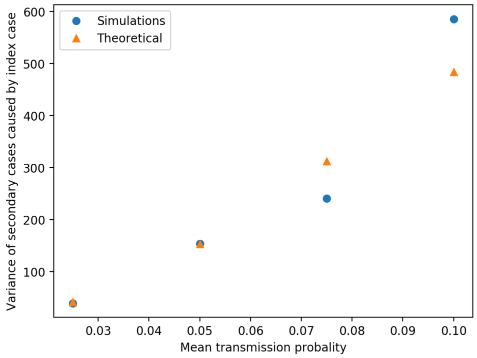

(5) $\alpha_i = 0.4$

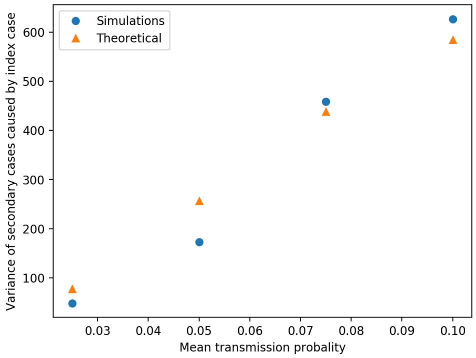

(6) $\alpha_i = 0.2$

Fig E. Comparison of the theoretical estimate for the variance of the individual reproduction number of an adult and the variance of the number of secondary cases per index case from simulations for the same individual for varying levels of infectiousness-related heterogeneity. Different values of α_i for the Truncated Gamma distribution considered for the individual transmission probability were tested.

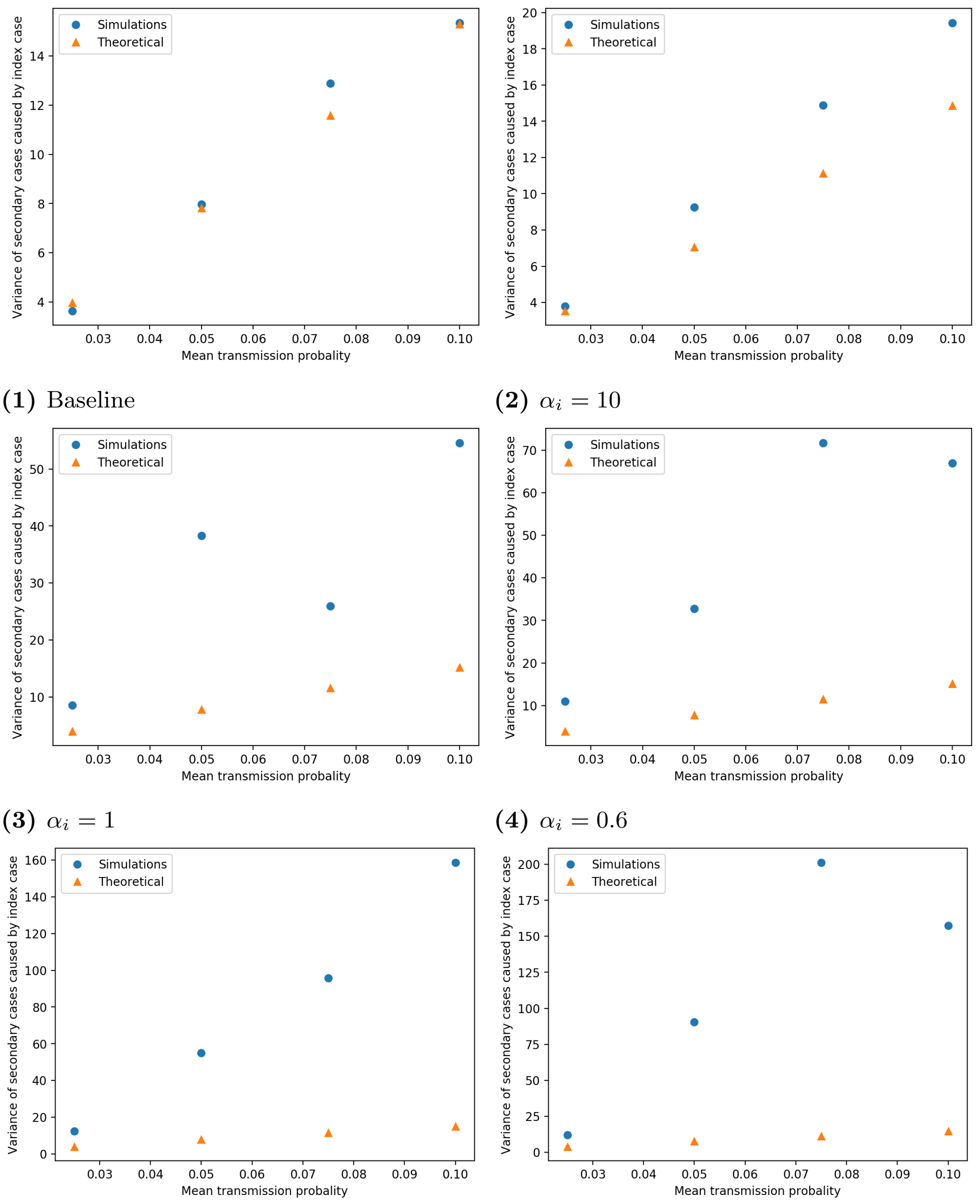

(1) Baseline

(2) $\alpha_i = 10$

(3) $\alpha_i = 1$

(4) $\alpha_i = 0.6$

(5) $\alpha_i = 0.4$

(6) $\alpha_i = 0.2$

Fig F. Comparison of the theoretical estimate for the variance of the individual reproduction number of an adult and the variance of the number of secondary cases per index case from simulations for the same individual for varying levels of contact-related heterogeneity. Different values of α_c for the Gamma distribution considered for the individual contact factor were tested.

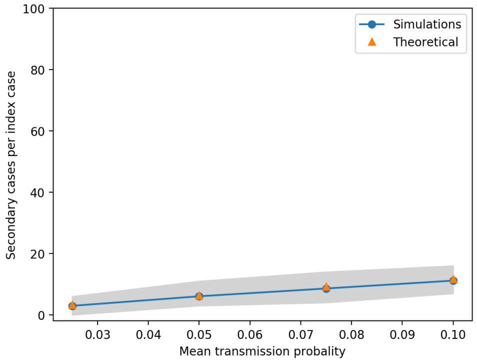

(1) Baseline

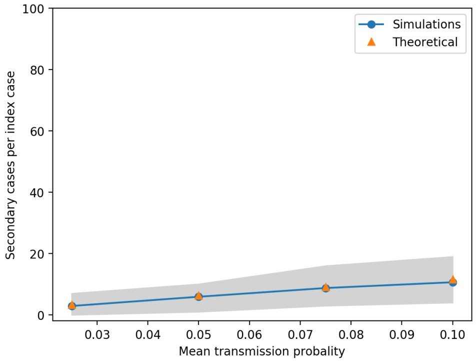

(2) $\alpha_i = 10$

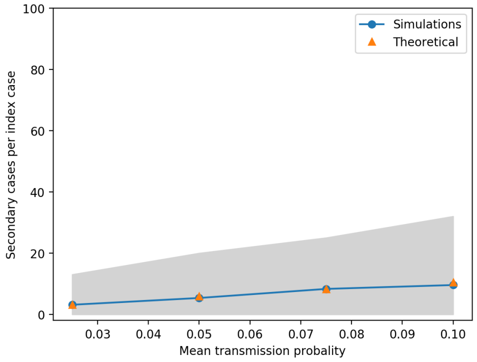

(3) $\alpha_i = 1$

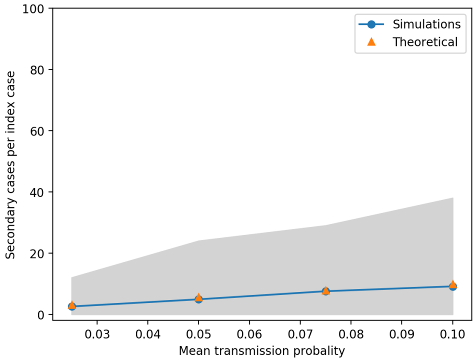

(4) $\alpha_i = 0.6$

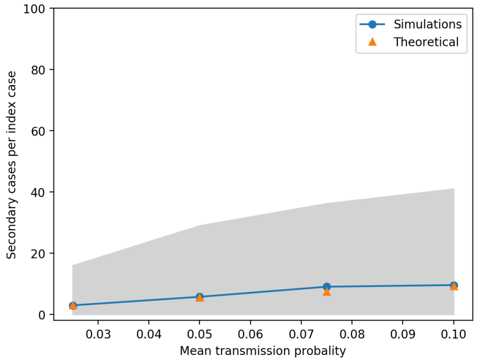

(5) $\alpha_i = 0.4$

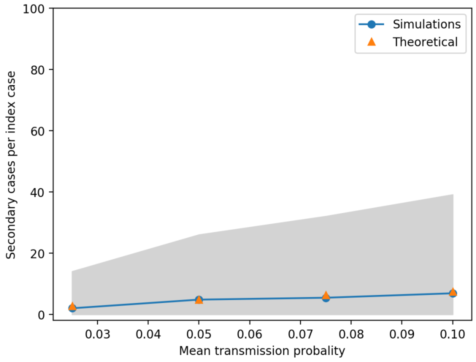

(6) $\alpha_i = 0.2$

Fig G. Comparison of the theoretical estimate for the mean individual reproduction number of a child and the mean number of secondary cases per index case from simulations for the same individual for varying levels of infectiousness-related heterogeneity. Different values of α_i for the Truncated Gamma distribution considered for the individual transmission probability were tested.

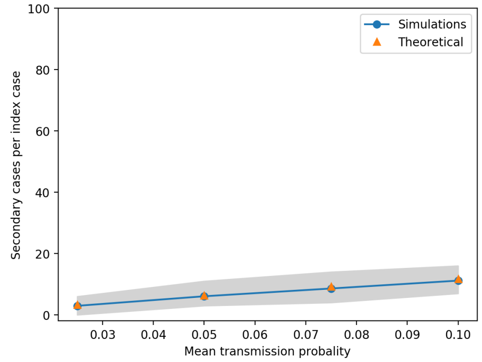

(1) Baseline

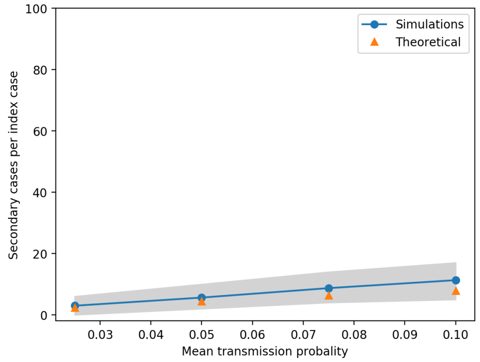

(2) $\alpha_c = 10$

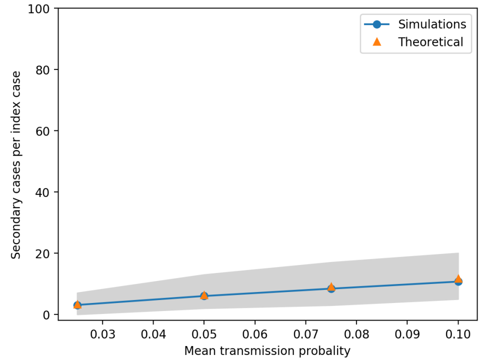

(3) $\alpha_c = 1$

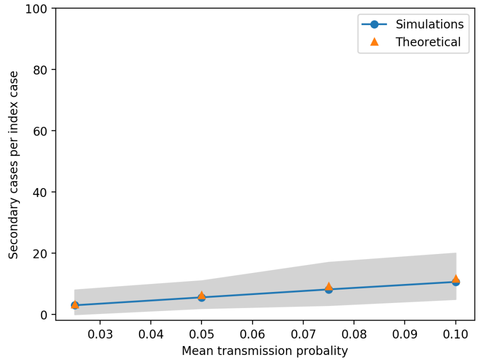

(4) $\alpha_c = 0.6$

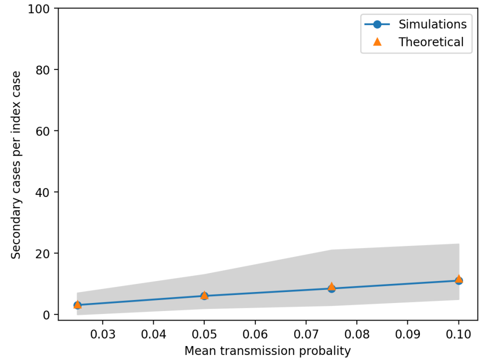

(5) $\alpha_c = 0.4$

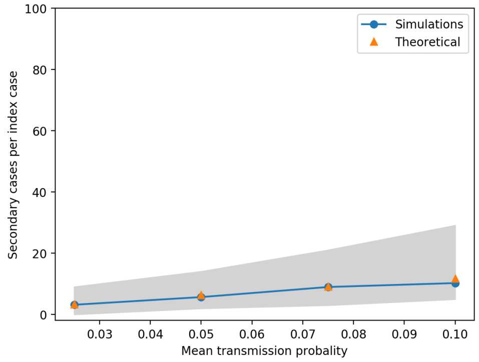

(6) $\alpha_c = 0.2$

Fig H. Comparison of the theoretical estimate for the mean individual reproduction number of a child and the mean number of secondary cases per index case from simulations for the same individual for varying levels of contact-related heterogeneity. Different values of α_c for the Gamma distribution considered for the individual contact factor were tested.

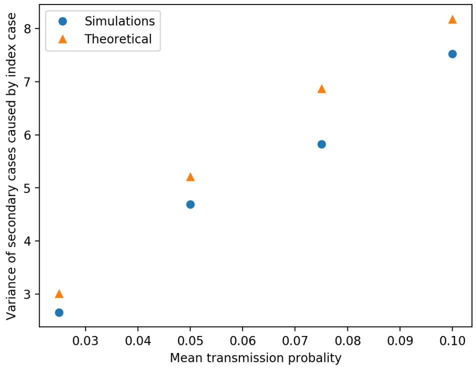

(1) Baseline

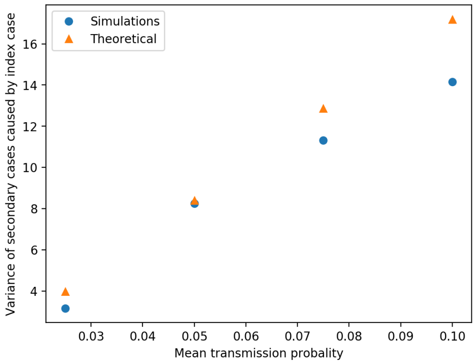

(2) $\alpha_i = 10$

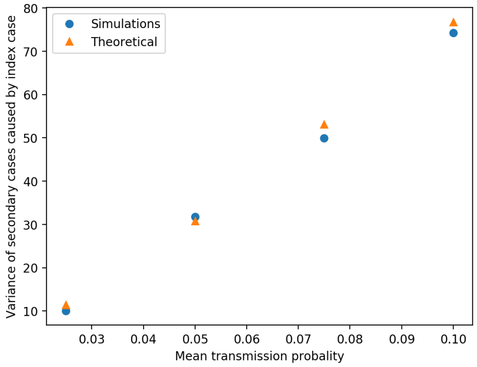

(3) $\alpha_i = 1$

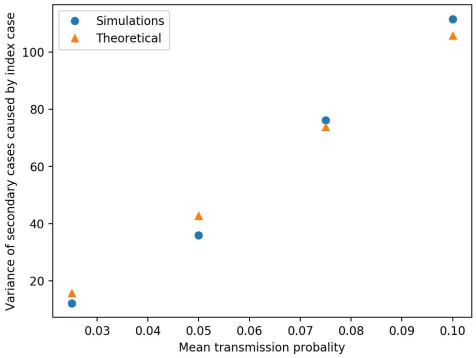

(4) $\alpha_i = 0.6$

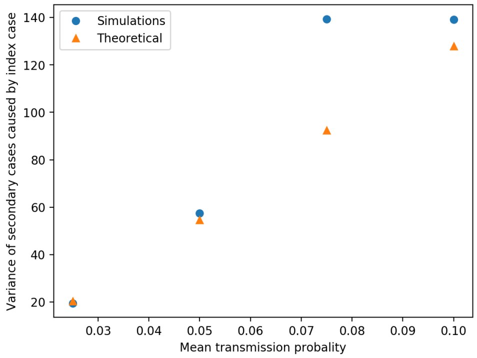

(5) $\alpha_i = 0.4$

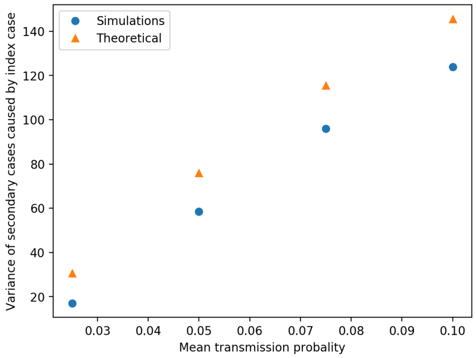

(6) $\alpha_i = 0.2$

Fig I. Comparison of the theoretical estimate for the variance of the individual reproduction number of a child and the variance of the number of secondary cases per index case from simulations for the same individual for varying levels of infectiousness-related heterogeneity. Different values of α_i for the Truncated Gamma distribution considered for the individual transmission probability were tested.

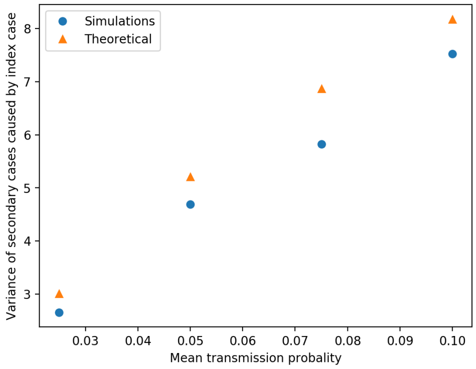

(1) Baseline

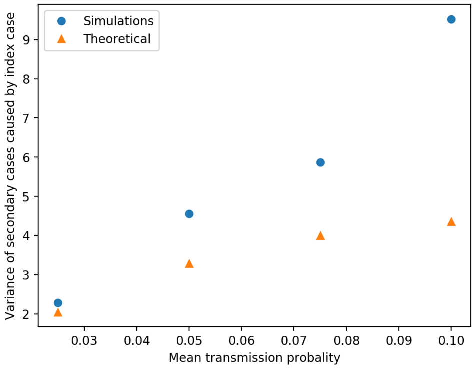

(2) $\alpha_i = 10$

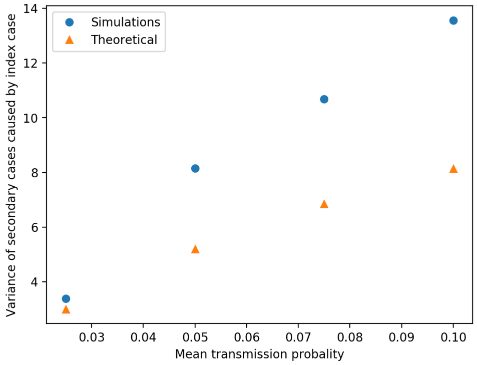

(3) $\alpha_i = 1$

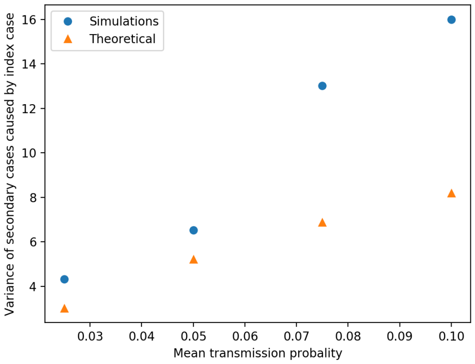

(4) $\alpha_i = 0.6$

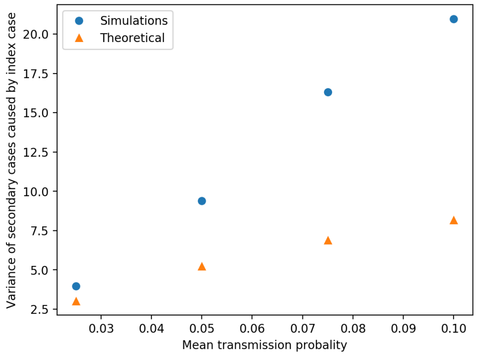

(5) $\alpha_i = 0.4$

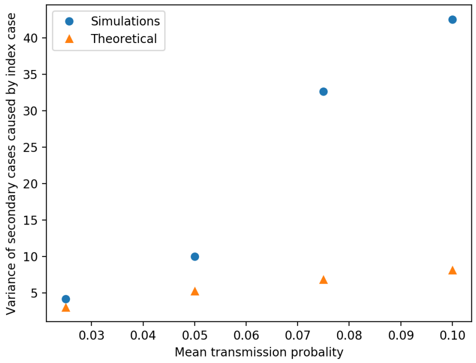

(6) $\alpha_i = 0.2$

Fig J. Comparison of the theoretical estimate for the variance of the individual reproduction number of a child and the variance of the number of secondary cases per index case from simulations for the same individual for varying levels of contact-related heterogeneity. Different values of α_c for the Gamma distribution considered for the individual contact factor were tested.

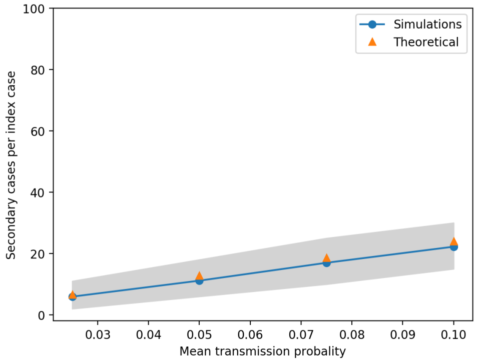

(1) Baseline

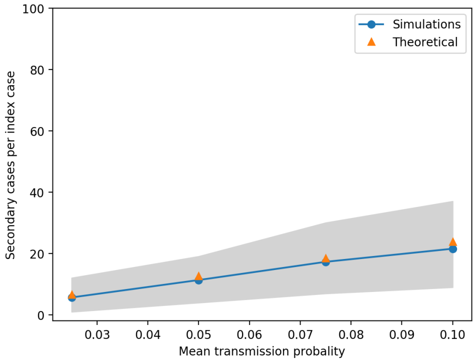

(2) $\alpha_i = 10$

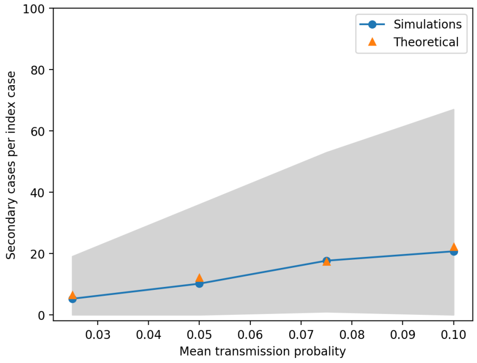

(3) $\alpha_i = 1$

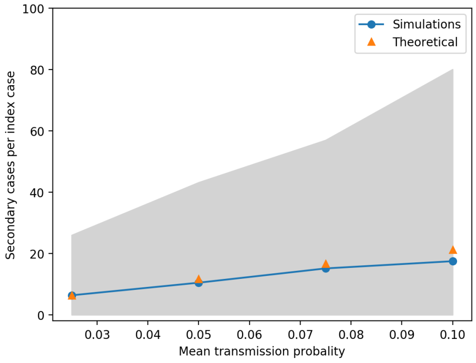

(4) $\alpha_i = 0.6$

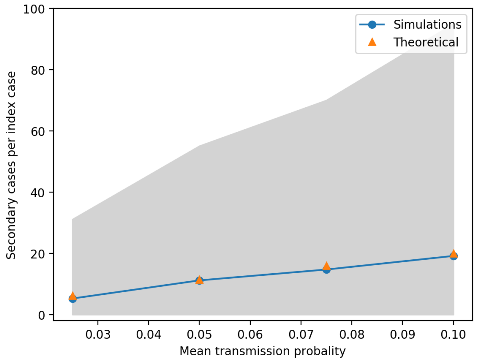

(5) $\alpha_i = 0.4$

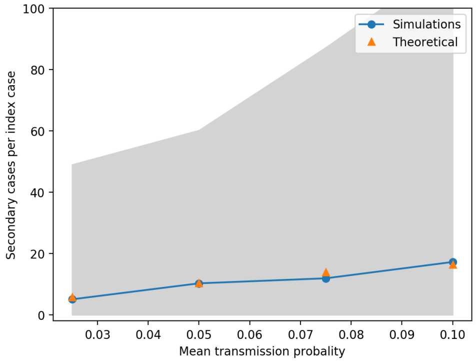

(6) $\alpha_i = 0.2$

Fig K. Comparison of the theoretical estimate for the mean individual reproduction number of an elderly person and the mean number of secondary cases per index case from simulations for the same individual for varying levels of infectiousness-related heterogeneity. Different values of α_i for the Truncated Gamma distribution considered for the individual transmission probability were tested.

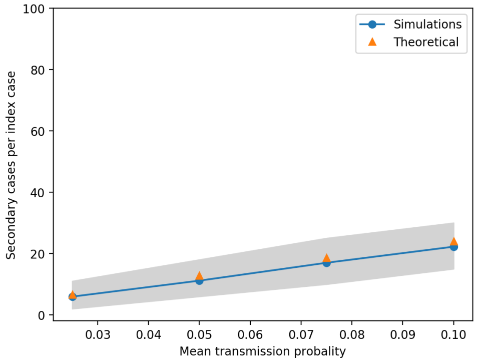

(1) Baseline

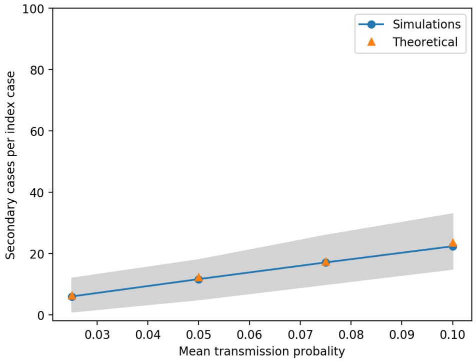

(2) $\alpha_c = 10$

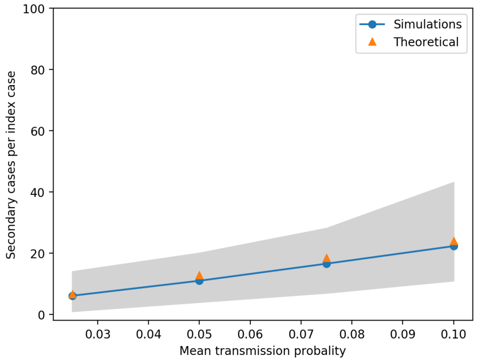

(3) $\alpha_c = 1$

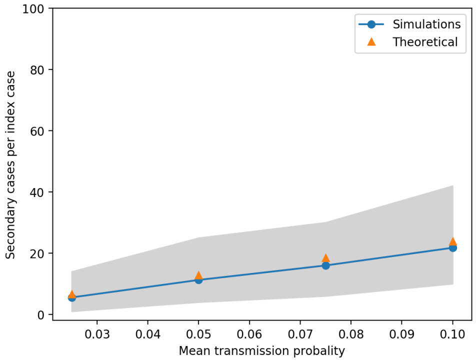

(4) $\alpha_c = 0.6$

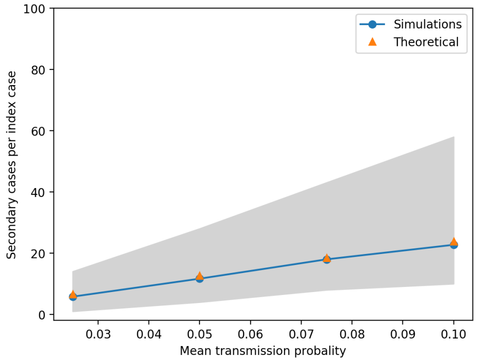

(5) $\alpha_c = 0.4$

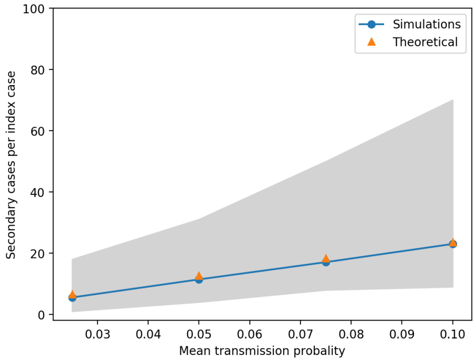

(6) $\alpha_c = 0.2$

Fig L. Comparison of the theoretical estimate for the mean individual reproduction number of an elderly person and the mean number of secondary cases per index case from simulations for the same individual for varying levels of contact-related heterogeneity. Different values of α_c for the Gamma distribution considered for the individual contact factor were tested.

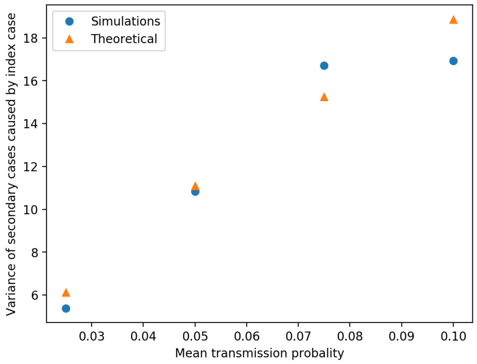

(1) Baseline

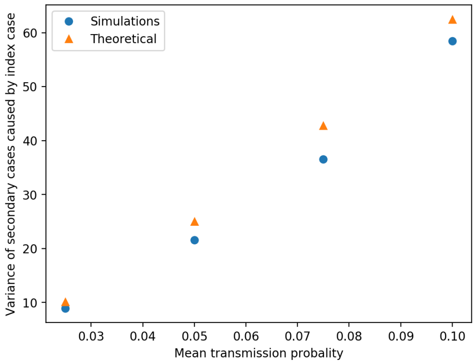

(2) $\alpha_i = 10$

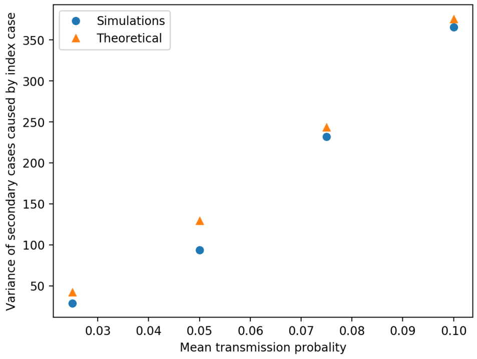

(3) $\alpha_i = 1$

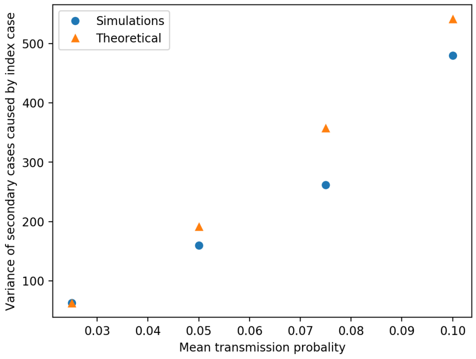

(4) $\alpha_i = 0.6$

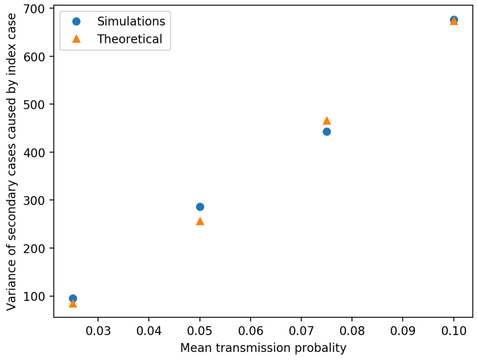

(5) $\alpha_i = 0.4$

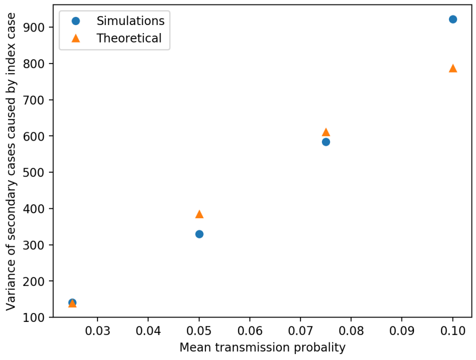

(6) $\alpha_i = 0.2$

Fig M. Comparison of the theoretical estimate for the variance of the individual reproduction number of an elderly person and the variance of the number of secondary cases per index case from simulations for the same individual for varying levels of infectiousness-related heterogeneity. Different values of α_i for the Truncated Gamma distribution considered for the individual transmission probability were tested.

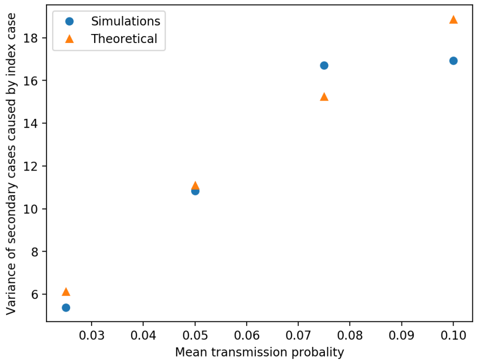

(1) Baseline

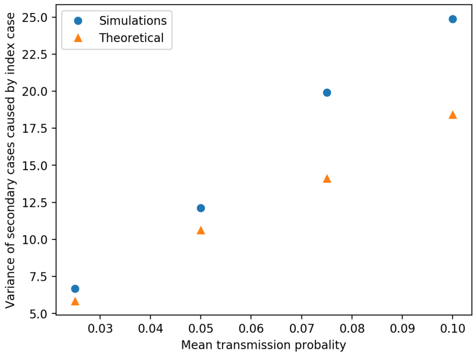

(2) $\alpha_c = 10$

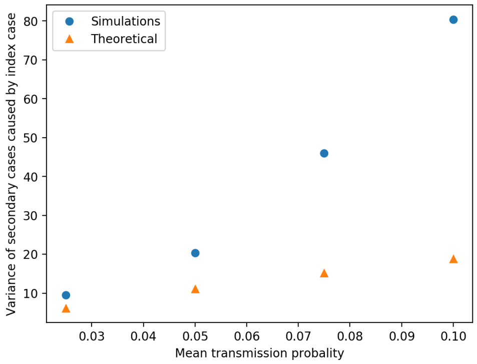

(3) $\alpha_c = 1$

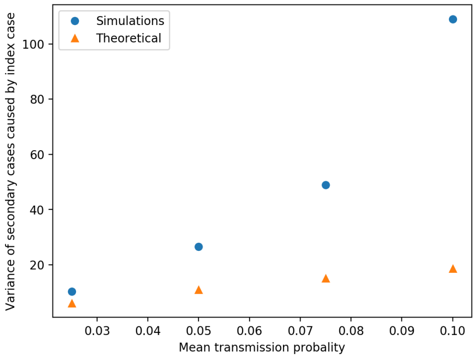

(4) $\alpha_c = 0.6$

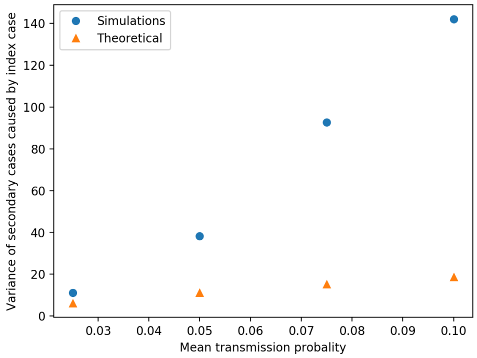

(5) $\alpha_c = 0.4$

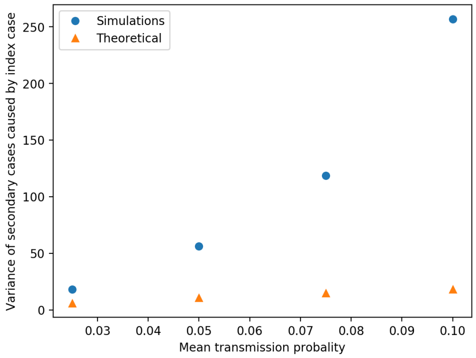

(6) $\alpha_c = 0.2$

Fig N. Comparison of the theoretical estimate for the variance of the individual reproduction number of an elderly person and the variance of the number of secondary cases per index case from simulations for the same individual for varying levels of contact-related heterogeneity. Different values of α_c for the Gamma distribution considered for the individual contact factor were tested.
