# Supplementary material for: Different forms of superspreading lead to different outcomes: Heterogeneity in infectiousness and contact behavior relevant for the case of SARS-CoV-2
Source: PLoS Comput Biol. 2022 Aug 22;18(8):e1009980. doi: 10.1371/journal.pcbi.1009980 (PMC9436127; doi:10.1371/journal.pcbi.1009980)
Supplement: S3 Text — (PDF) [file pcbi.1009980.s003.pdf]

S3 Text. Sensitivity analysis.

Elise J. Kuylen[1,2*], Andrea Torneri[1], Lander Willem[1], Pieter J. K. Libin[2,3,4], Steven Abrams[2,5], Pietro Coletti[2], Nicolas Franco[2,6], Frederik Verelst[1], Philippe Beutels[1,7], Jori Liesenborgs[8], Niel Hens[1,2]

1 Centre for Health Economic Research and Modeling Infectious Diseases, University of Antwerp, Antwerp, Belgium
2 Data Science Institute, I-BioStat, Hasselt University, Hasselt, Belgium
3 Artificial Intelligence Lab, Vrije Universiteit Brussel, Brussels, Belgium
4 Rega Institute for Medical Research, Clinical and Epidemiological Virology, University of Leuven, Leuven, Belgium
5 Global Health Institute, University of Antwerp, Antwerp, Belgium
6 Namur Institute for Complex Systems, Department of Mathematics, University of Namur, Namur, Belgium
7 School of Public Health and Community Medicine, The University of New South Wales, Sydney, NSW, Australia
8 Expertise Centre for Digital Media, Hasselt University - transnational University Limburg, Hasselt, Belgium

* elise.kuylen@uantwerp.be

To conduct the sensitivity analysis, we varied the value of two parameters: the number of infected seeds at the beginning of the simulation and the mean transmission probability. In our main analysis, we introduced one infected individual in the population at the beginning of each simulation. For the sensitivity analysis we introduced 10, 50 and 100 infected seeds at the beginning of the simulation. For the mean transmission probability we used 0.08 in our main analysis, and additionally tested the values 0.06 and 0.10 for our sensitivity analysis.

We then checked the behavior of the following outcomes for the scenarios without interventions: the extinction probability (Fig A), the mean attack rate after 200 days (Fig B), the mean size of the peak (Fig C), the mean day on which the peak is reached (Fig D), the mean estimated herd immunity threshold (Fig E), the mean day on which the herd immunity threshold is reached (Fig F), and the mean day on which the last transmission event is observed (Fig G).

We found that, for most outcomes, varying either the number of infected seeds at the beginning of the simulation or the mean transmission probability, led to the same trends being observed when α_i or α_c decreased.

The only exception we observed, was that, with a mean transmission probability of 0.1, the mean size of the peak decreased slightly (instead of increasing) when α_c was decreased from 10 to 0.2 (see Fig C4).

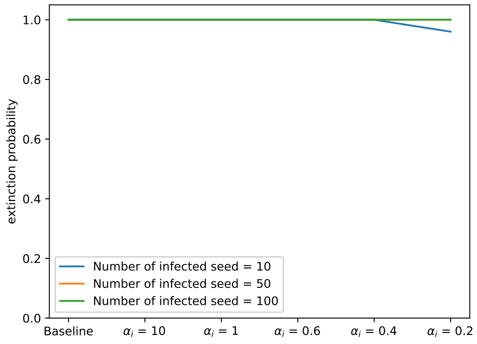

(1) Impact of introducing different numbers of infected seeds at the beginning of the simulation when varying α_i for the Truncated Gamma distribution considered for the individual transmission probability.

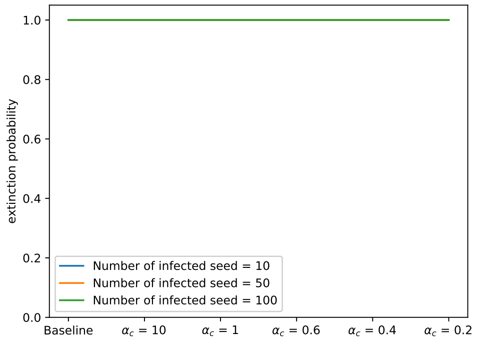

(2) Impact of introducing different numbers of infected seeds at the beginning of the simulation when varying α_c for the Gamma distribution considered for the individual contact factor.

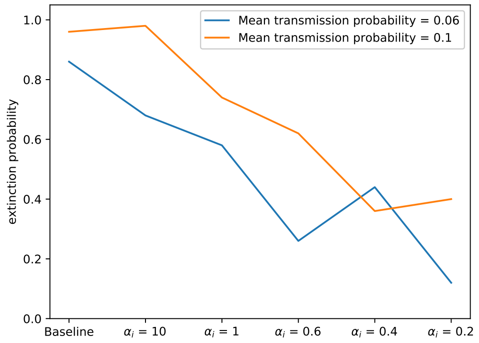

(3) Impact of different values for the mean transmission probability when varying α_i for the Truncated Gamma distribution considered for the individual transmission probability.

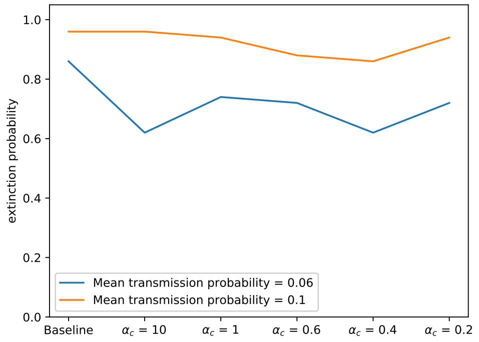

(4) Impact of different values for the mean transmission probability when varying α_c for the Gamma distribution considered for the individual contact factor.

Fig A. Impact of different values for the number of infected seeds at the beginning of the simulation (panel 1 and 2) **and for the mean transmission probability** (panel 3 and 4) **on the extinction probability.** The extinction threshold was set at 50 cases.

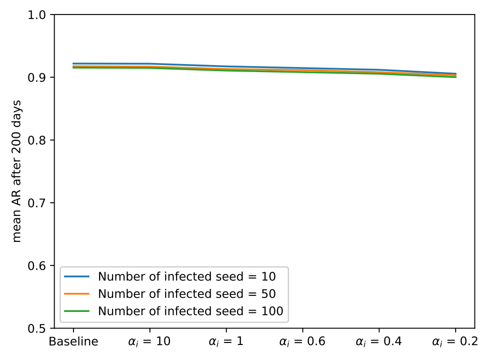

(1) Impact of introducing different numbers of infected seeds at the beginning of the simulation when varying α_i for the Truncated Gamma distribution considered for the individual transmission probability.

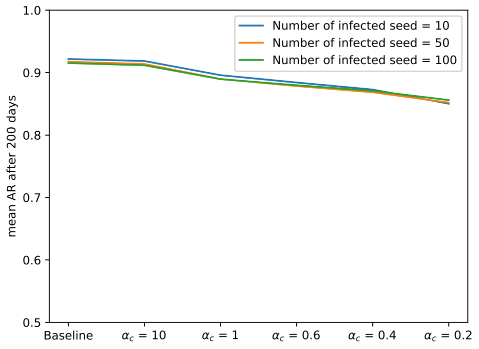

(2) Impact of introducing different numbers of infected seeds at the beginning of the simulation when varying α_c for the Gamma distribution considered for the individual contact factor.

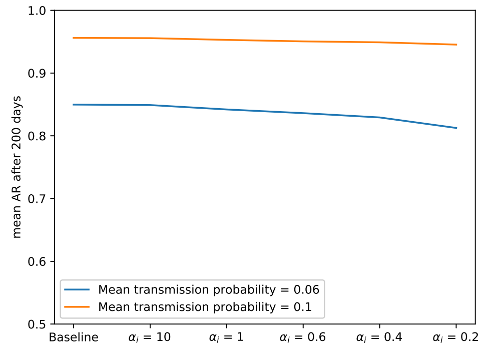

(3) Impact of different values for the mean transmission probability when varying α_i for the Truncated Gamma distribution considered for the individual transmission probability.

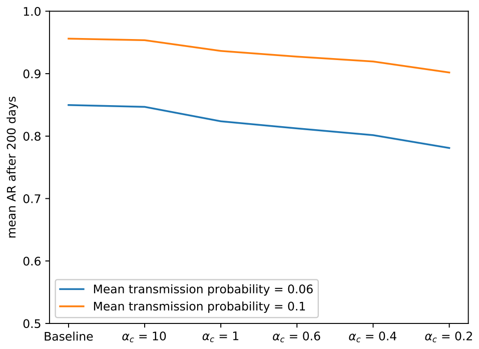

(4) Impact of different values for the mean transmission probability when varying α_c for the Gamma distribution considered for the individual contact factor.

Fig B. Impact of different values for the number of infected seeds at the beginning of the simulation (panel 1 and 2) **and for the mean transmission probability** (panel 3 and 4) **on the mean attack rate over 200 days.** Simulation runs which led to extinction (i.e. those that produced less than 50 cases) were excluded.

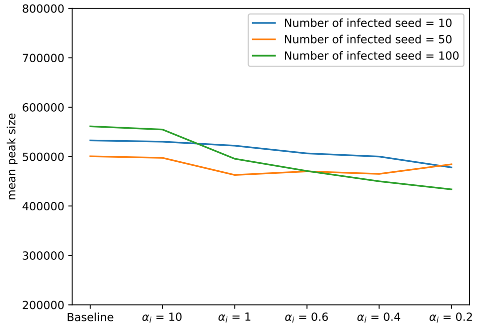

(1) Impact of introducing different numbers of infected seeds at the beginning of the simulation when varying α_i for the Truncated Gamma distribution considered for the individual transmission probability.

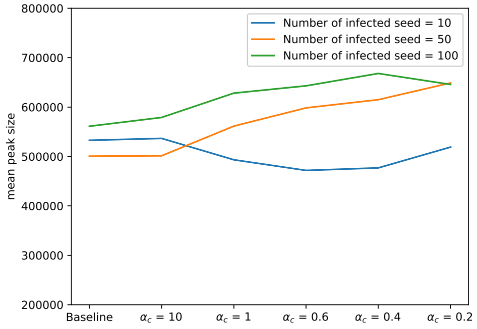

(2) Impact of introducing different numbers of infected seeds at the beginning of the simulation when varying α_c for the Gamma distribution considered for the individual contact factor.

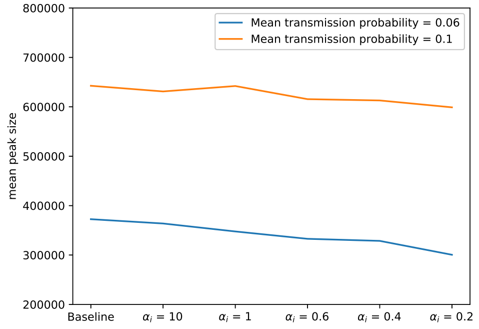

(3) Impact of different values for the mean transmission probability when varying α_i for the Truncated Gamma distribution considered for the individual transmission probability.

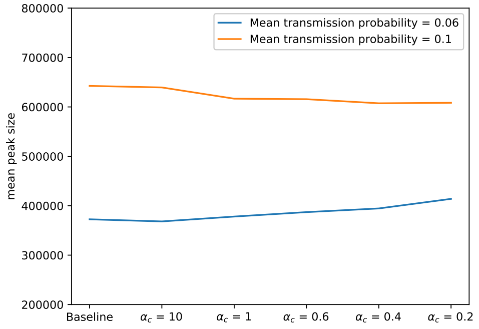

(4) Impact of different values for the mean transmission probability when varying α_c for the Gamma distribution considered for the individual contact factor.

Fig C. Impact of different values for the number of infected seeds at the beginning of the simulation (panel 1 and 2) **and for the mean transmission probability** (panel 3 and 4) **on the mean size of the peak.** Simulation runs which led to extinction (i.e. those that produced less than 50 cases) were excluded.

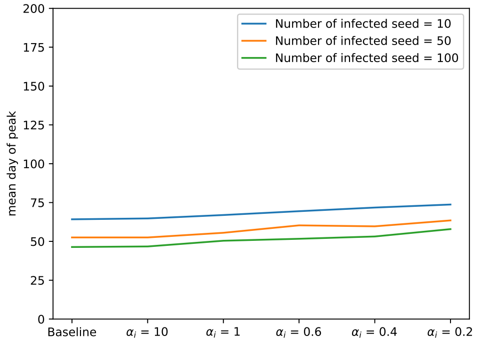

(1) Impact of introducing different numbers of infected seeds at the beginning of the simulation when varying α_i for the Truncated Gamma distribution considered for the individual transmission probability.

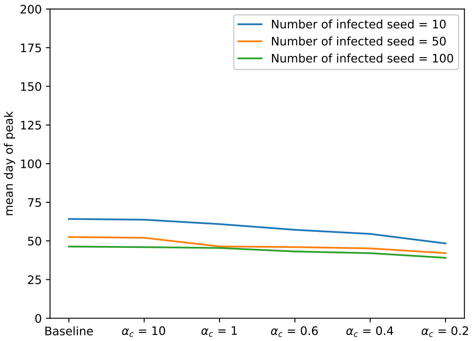

(2) Impact of introducing different numbers of infected seeds at the beginning of the simulation when varying α_c for the Gamma distribution considered for the individual contact factor.

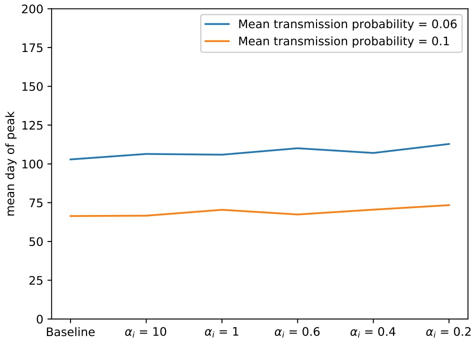

(3) Impact of different values for the mean transmission probability when varying α_i for the Truncated Gamma distribution considered for the individual transmission probability.

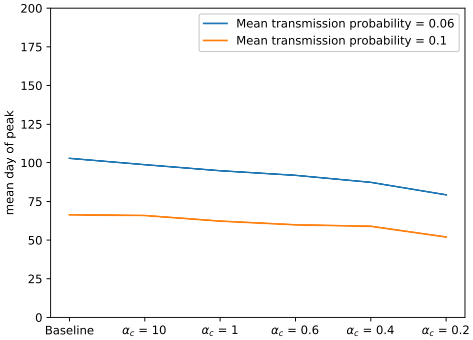

(4) Impact of different values for the mean transmission probability when varying α_c for the Gamma distribution considered for the individual contact factor.

Fig D. Impact of different values for the number of infected seeds at the beginning of the simulation (panel 1 and 2) **and for the mean transmission probability** (panel 3 and 4) **on the mean day on which the peak is reached.** Simulation runs which led to extinction (i.e. those that produced less than 50 cases) were excluded.

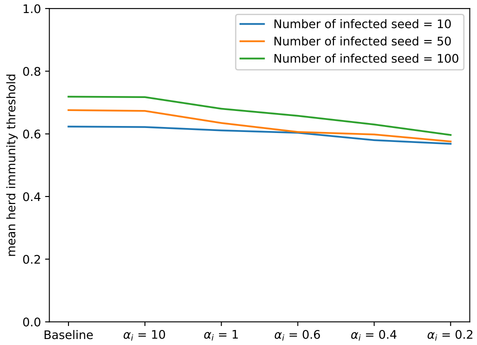

(1) Impact of introducing different numbers of infected seeds at the beginning of the simulation when varying α_i for the Truncated Gamma distribution considered for the individual transmission probability.

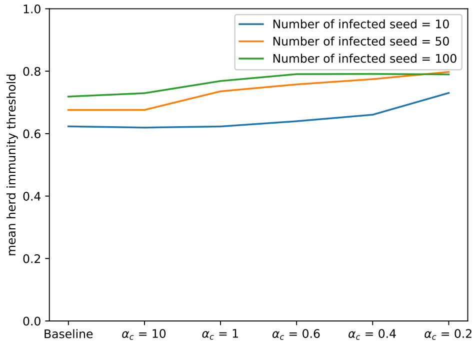

(2) Impact of introducing different numbers of infected seeds at the beginning of the simulation when varying α_c for the Gamma distribution considered for the individual contact factor.

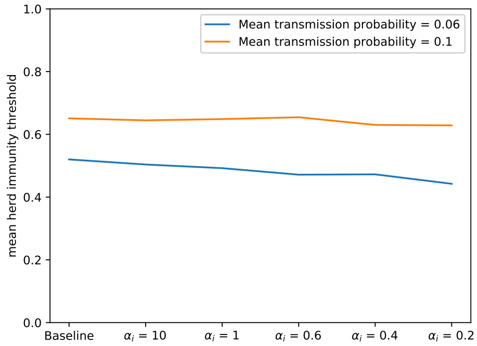

(3) Impact of different values for the mean transmission probability when varying α_i for the Truncated Gamma distribution considered for the individual transmission probability.

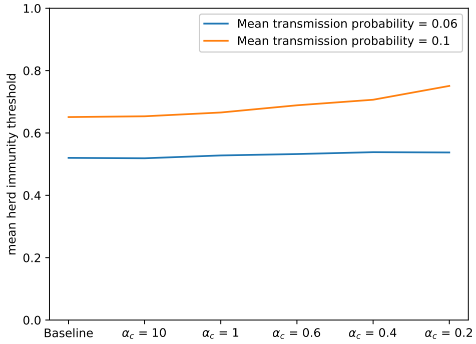

(4) Impact of different values for the mean transmission probability when varying α_c for the Gamma distribution considered for the individual contact factor.

Fig E. Impact of different values for the number of infected seeds at the beginning of the simulation (panel 1 and 2) **and for the mean transmission probability** (panel 3 and 4) **on the mean estimated herd immunity threshold.** Simulation runs which led to extinction (i.e. those that produced less than 50 cases) were excluded.

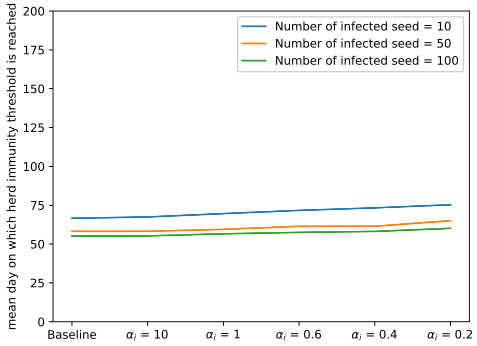

(1) Impact of introducing different numbers of infected seeds at the beginning of the simulation when varying α_i for the Truncated Gamma distribution considered for the individual transmission probability.

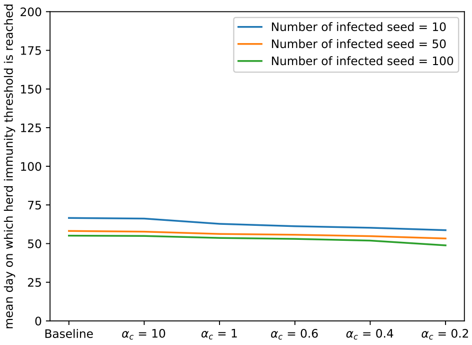

(2) Impact of introducing different numbers of infected seeds at the beginning of the simulation when varying α_c for the Gamma distribution considered for the individual contact factor.

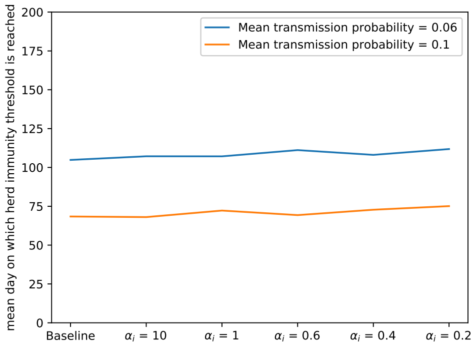

(3) Impact of different values for the mean transmission probability when varying α_i for the Truncated Gamma distribution considered for the individual transmission probability.

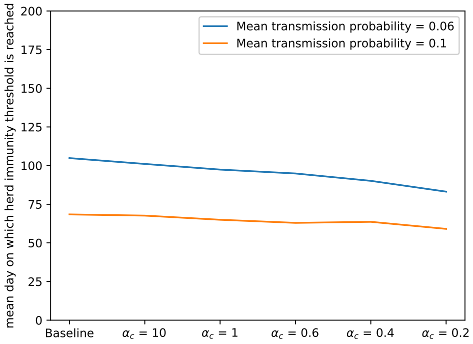

(4) Impact of different values for the mean transmission probability when varying α_c for the Gamma distribution considered for the individual contact factor.

Fig F. Impact of different values for the number of infected seeds at the beginning of the simulation (panel 1 and 2) **and for the mean transmission probability** (panel 3 and 4) **on the mean day on which the herd immunity threshold is reached.** Simulation runs which led to extinction (i.e. those that produced less than 50 cases) were excluded.

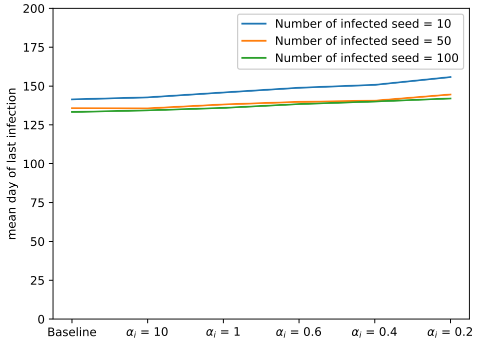

(1) Impact of introducing different numbers of infected seeds at the beginning of the simulation when varying α_i for the Truncated Gamma distribution considered for the individual transmission probability.

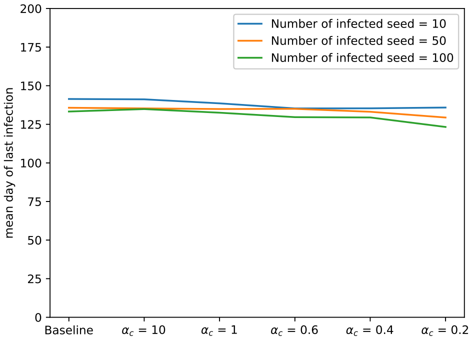

(2) Impact of introducing different numbers of infected seeds at the beginning of the simulation when varying α_c for the Gamma distribution considered for the individual contact factor.

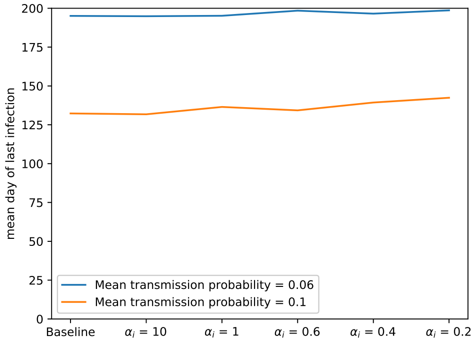

(3) Impact of different values for the mean transmission probability when varying α_i for the Truncated Gamma distribution considered for the individual transmission probability.

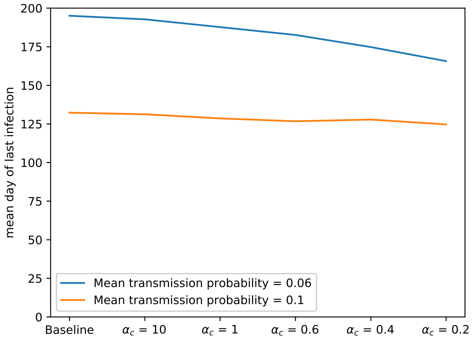

(4) Impact of different values for the mean transmission probability when varying α_c for the Gamma distribution considered for the individual contact factor.

Fig G. Impact of different values for the number of infected seeds at the beginning of the simulation (panel 1 and 2) **and for the mean transmission probability** (panel 3 and 4) **on the mean day on which the last transmission event is observed.** Simulation runs which led to extinction (i.e. those that produced less than 50 cases) were excluded.
