# Supplementary material for: Different forms of superspreading lead to different outcomes: Heterogeneity in infectiousness and contact behavior relevant for the case of SARS-CoV-2
Source: PLoS Comput Biol. 2022 Aug 22;18(8):e1009980. doi: 10.1371/journal.pcbi.1009980 (PMC9436127; doi:10.1371/journal.pcbi.1009980)
Supplement: S4 Text — (PDF) [file pcbi.1009980.s004.pdf]

S4 Text. Varying α_i and α_c together.

Elise J. Kuylen[1,2*], Andrea Torneri[1], Lander Willem[1], Pieter J. K. Libin[2,3,4], Steven Abrams[2,5], Pietro Coletti[2], Nicolas Franco[2,6], Frederik Verelst[1], Philippe Beutels[1,7], Jori Liesenborgs[8], Niel Hens[1,2]

1 Centre for Health Economic Research and Modeling Infectious Diseases, University of Antwerp, Antwerp, Belgium
2 Data Science Institute, I-BioStat, Hasselt University, Hasselt, Belgium
3 Artificial Intelligence Lab, Vrije Universiteit Brussel, Brussels, Belgium
4 Rega Institute for Medical Research, Clinical and Epidemiological Virology, University of Leuven, Leuven, Belgium
5 Global Health Institute, University of Antwerp, Antwerp, Belgium
6 Namur Institute for Complex Systems, Department of Mathematics, University of Namur, Namur, Belgium
7 School of Public Health and Community Medicine, The University of New South Wales, Sydney, NSW, Australia
8 Expertise Centre for Digital Media, Hasselt University - transnational University Limburg, Hasselt, Belgium

* elise.kuylen@uantwerp.be

To explore the effect of different combinations of heterogeneity in infectiousness and heterogeneity in contacts, we ran simulations for a grid of configurations. We used a simple Latin Hypercube Sample to design a grid of 64 combinations of α_i and α_c. For each of these combinations, we ran 50 simulations, using the parameters as described in the main text under subsection "Superspreading effects in the absence of interventions".

We verified that the mean R_0 remained relatively stable over all scenarios. This can be observed in Fig A1.

Next, we calculated the mean P_{80} for each scenario. This is shown in Fig A2. It can be observed that α_i has a larger impact on P_{80} than α_c. As P_{80} for SARS-CoV-2 is estimated to be about 0.10, α_i could be estimated between 0.25 and 0.35, depending on the value of α_c, which could be extracted from contact surveys conducted during the pandemic.

We then investigated the effect of combinations of values for α_i and α_c on epidemiological outcomes. First, we looked at the probability of extinction. In accordance with our analysis of the scenarios in the main text, we observed the final sizes of the epidemics in all scenarios to formulate an 'extinction threshold', which all outbreaks either remained below or exceeded by far. For the grid of scenarios we tested here, we set the extinction threshold at 50 cases. Based on this threshold, we calculated the fraction of simulations runs per scenario that went extinct after less than 50 cases (shown in Fig B1). It is clear that α_i has a larger impact on the extinction probability than α_c does. In fact, it is not clear what exactly the influence of α_c for this outcome is.

Conversely, α_c seems to be the most influential parameter for the attack rate over the entire epidemic (as shown in Fig B2). However, lowering α_i as well as α_c both have the effect of decreasing the attack rate.

The size of the peak seems to be mainly influenced by α_i, while its timing is impacted more by α_c. However, both effects are not as clear as the effect on the attack rate. This is shown in Fig B3–4.

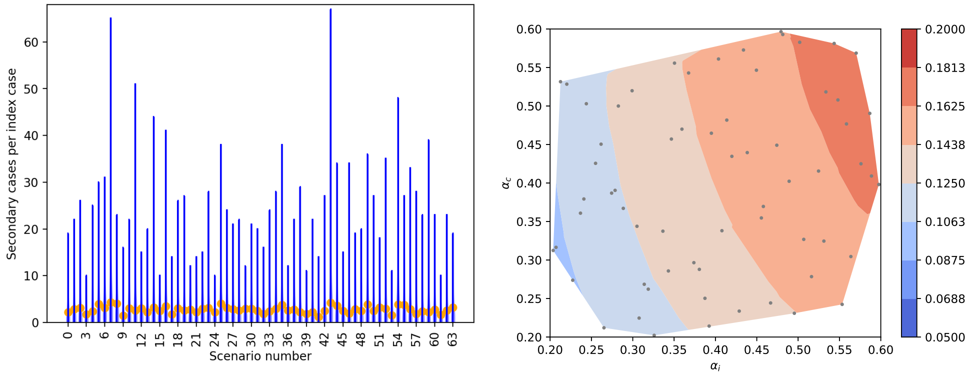

(1) Mean number of secondary cases per index case.

(2) P_{80}

Fig A. Mean number of secondary cases per index case and P_{80} for different combinations of α_i and α_c.

A similar effect can be seen when observing the herd immunity threshold and the day on which the last transmission event occurs. The herd immunity threshold – which, as before, is defined as the fraction of the population that is no longer susceptible on the last day for which $R_t \geq 1$ – is mainly influenced by α_i, while the timing of the end of the epidemic (i.e. the day on which the last transmission event is observed) is mainly influenced by α_c. This is shown in Fig B5–6.

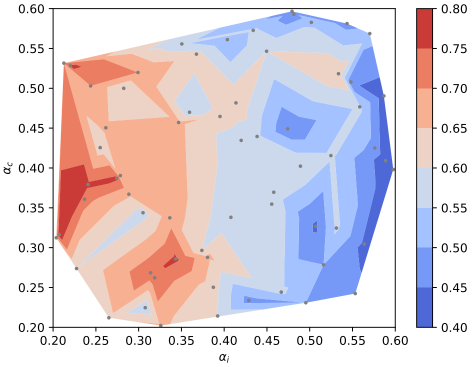

(1) Fraction of simulation runs for which extinction occurs (threshold = 50 cases).

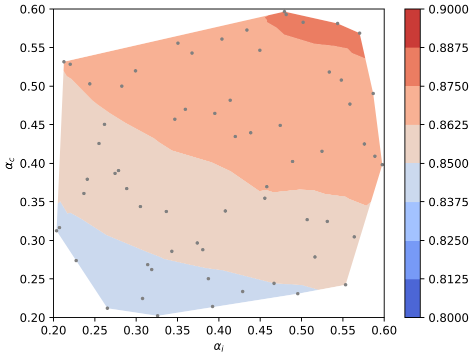

(2) Attack rate over 200 days. Simulations in which extinction occurs were excluded.

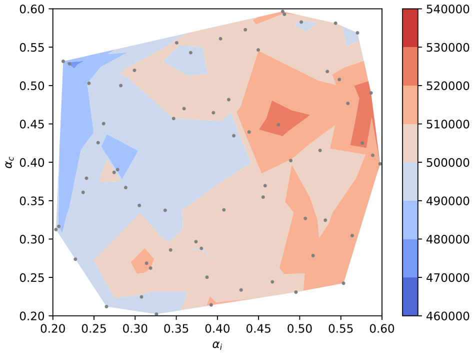

(3) Peak size (maximum number of new cases in 1 day). Simulations in which extinction occurs were excluded.

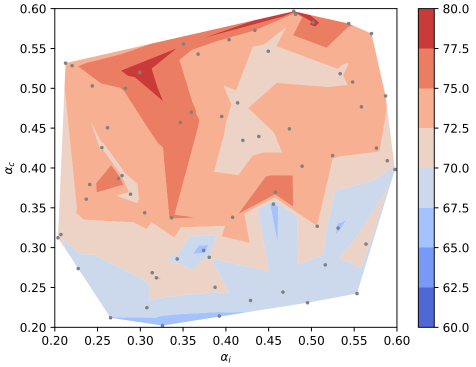

(4) Day on which the peak occurs. Simulations in which extinction occurs were excluded.

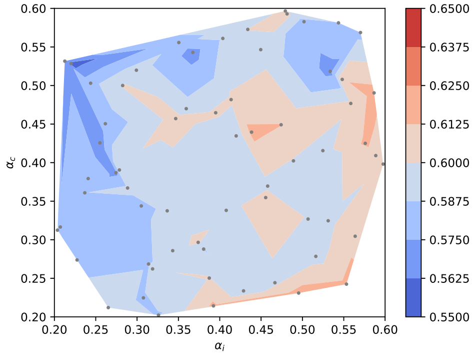

(5) Herd immunity threshold (fraction of the population that is no longer immune on the last day for which $R_t \geq 1$). Simulations in which extinction occurs were excluded.

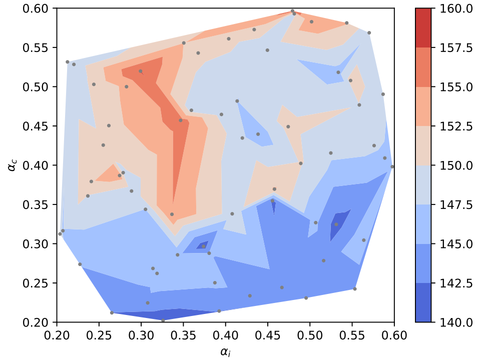

(6) Day on which the last transmission event is observed. Simulations in which extinction occurs were excluded.

Fig B. Extinction probability, attack rate, peak size and timing, herd immunity threshold, and day on which the last transmission event is observed for different combinations of α_i and α_c. Grey dots represent the coordinates of the points in the grid that were tested.
