# Supplementary material for: Different forms of superspreading lead to different outcomes: Heterogeneity in infectiousness and contact behavior relevant for the case of SARS-CoV-2
Source: PLoS Comput Biol. 2022 Aug 22;18(8):e1009980. doi: 10.1371/journal.pcbi.1009980 (PMC9436127; doi:10.1371/journal.pcbi.1009980)
Supplement: S1 File — (PDF) [file pcbi.1009980.s005.pdf]

S1 File. Supplementary figures.

Elise J. Kuylen[1,2*], Andrea Torneri[1], Lander Willem[1], Pieter J. K. Libin[2,3,4], Steven Abrams[2,5], Pietro Coletti[2], Nicolas Franco[2,6], Frederik Verelst[1], Philippe Beutels[1,7], Jori Liesenborgs[8], Niel Hens[1,2]

1 Centre for Health Economic Research and Modeling Infectious Diseases, University of Antwerp, Antwerp, Belgium
2 Data Science Institute, I-BioStat, Hasselt University, Hasselt, Belgium
3 Artificial Intelligence Lab, Vrije Universiteit Brussel, Brussels, Belgium
4 Rega Institute for Medical Research, Clinical and Epidemiological Virology, University of Leuven, Leuven, Belgium
5 Global Health Institute, University of Antwerp, Antwerp, Belgium
6 Namur Institute for Complex Systems, Department of Mathematics, University of Namur, Namur, Belgium
7 School of Public Health and Community Medicine, The University of New South Wales, Sydney, NSW, Australia
8 Expertise Centre for Digital Media, Hasselt University - transnational University Limburg, Hasselt, Belgium

* elise.kuylen@uantwerp.be

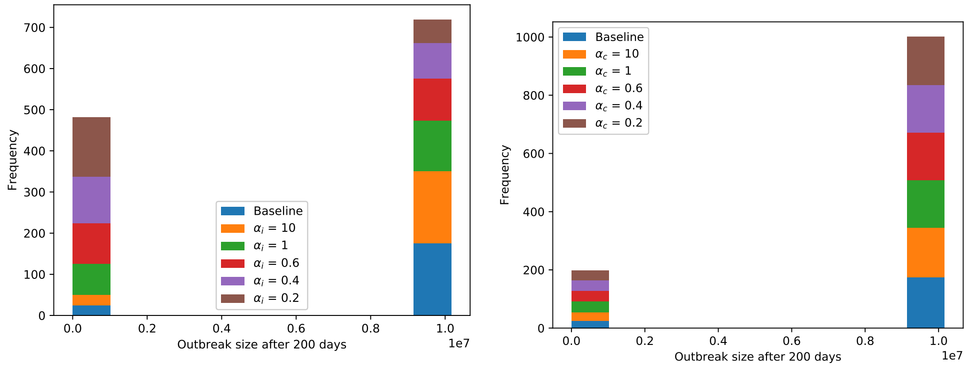

(1) Varying α_i for the Truncated Gamma distribution considered the individual transmission probability.

(2) Varying α_c for the Gamma distribution considered for the individual contact factor.

Fig A. Histograms of final sizes for the different scenarios regarding infectiousness-related and contact-related heterogeneity, without interventions.

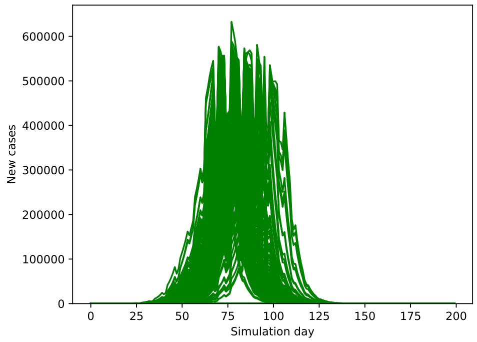

(1) Baseline

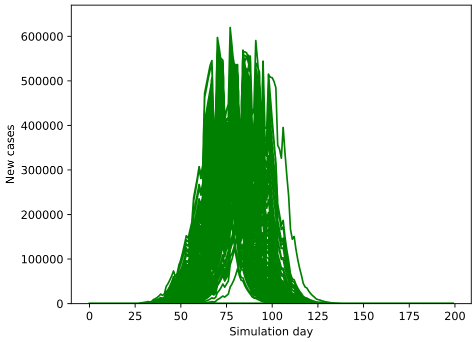

(2) $\alpha_i = 10$

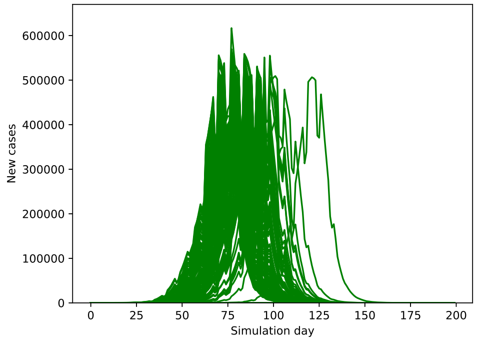

(3) $\alpha_i = 1$

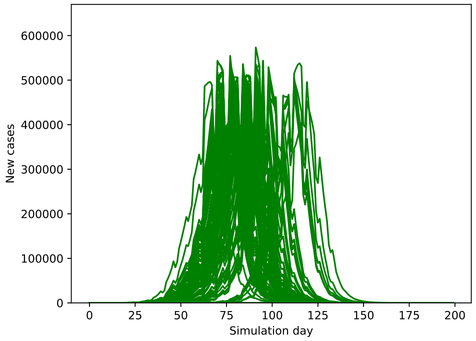

(4) $\alpha_i = 0.6$

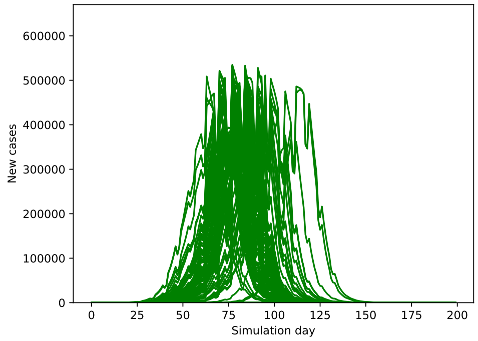

(5) $\alpha_i = 0.4$

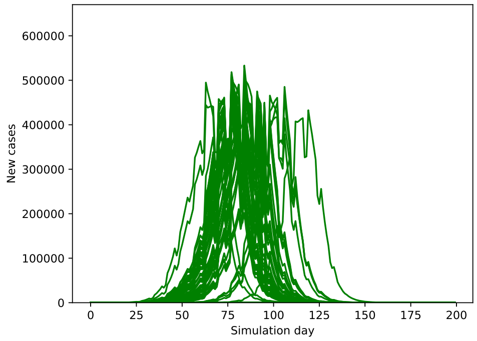

(6) $\alpha_i = 0.2$

Fig B. Evolution of the number of new cases per day for different values of α_i for the Truncated Gamma distribution considered for the individual transmission probability, for the scenario without interventions.

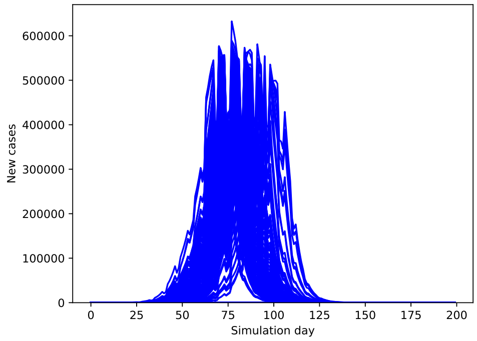

(1) Baseline

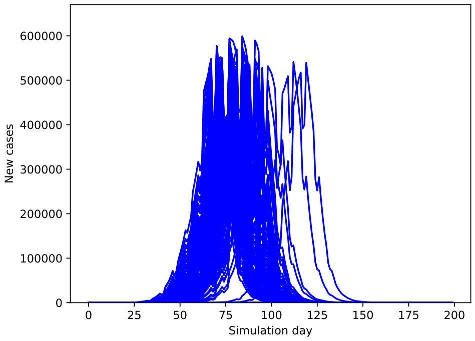

(2) $\alpha_c = 10$

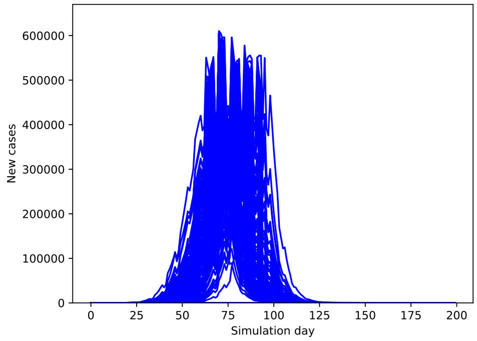

(3) $\alpha_c = 1$

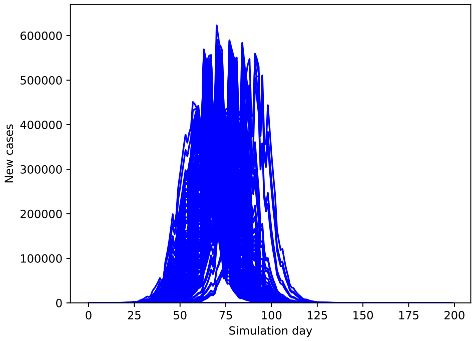

(4) $\alpha_c = 0.6$

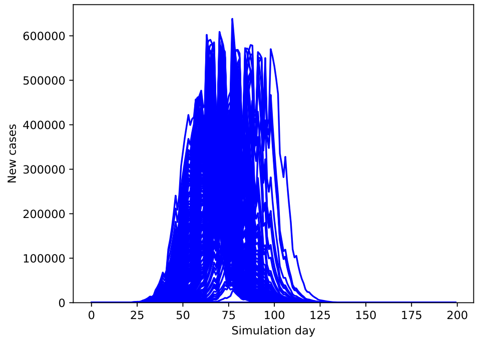

(5) $\alpha_c = 0.4$

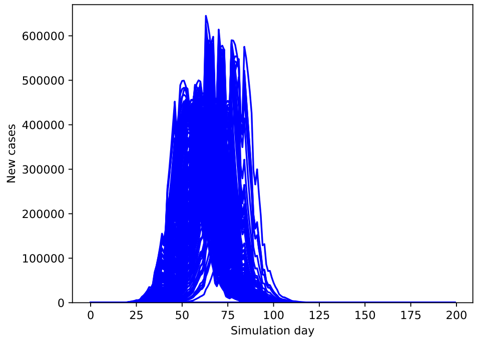

(6) $\alpha_c = 0.2$

Fig C. Evolution of the number of new cases per day for different values of α_c for the Gamma distribution considered for the individual contact factor, for the scenario without interventions.

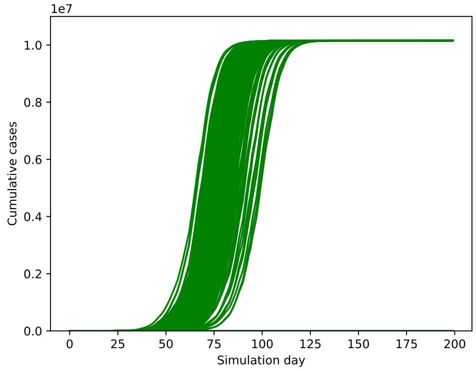

(1) Baseline

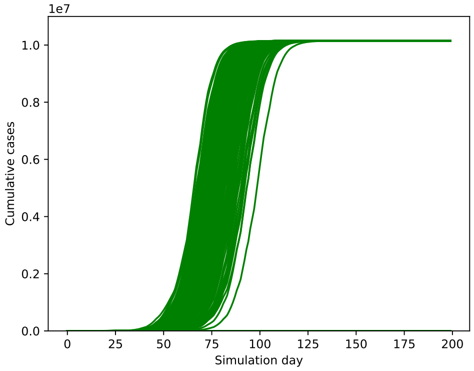

(2) $\alpha_i = 10$

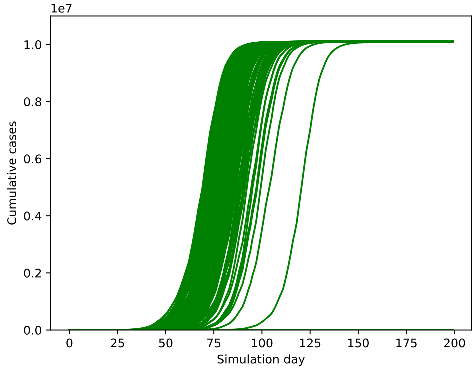

(3) $\alpha_i = 1$

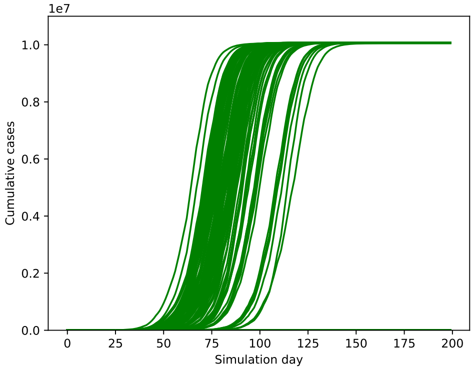

(4) $\alpha_i = 0.6$

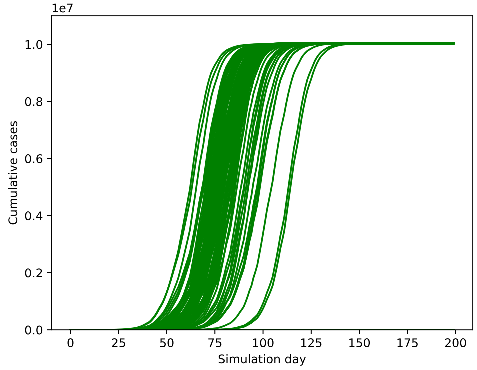

(5) $\alpha_i = 0.4$

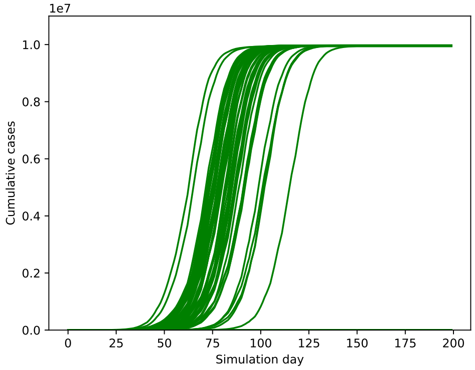

(6) $\alpha_i = 0.2$

Fig D. Evolution of the number of cumulative cases per day for different values of α_i for the Truncated Gamma distribution considered for the individual transmission probability, for the scenario without interventions.

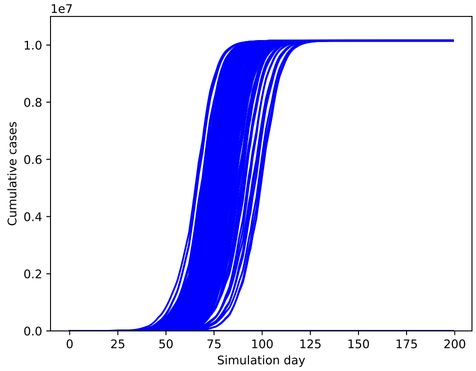

(1) Baseline

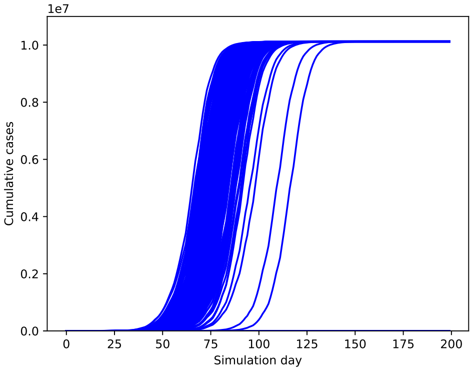

(2) $\alpha_c = 10$

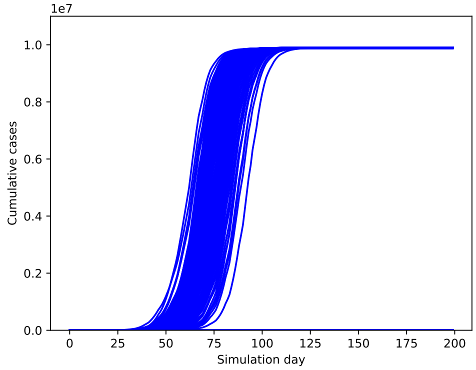

(3) $\alpha_c = 1$

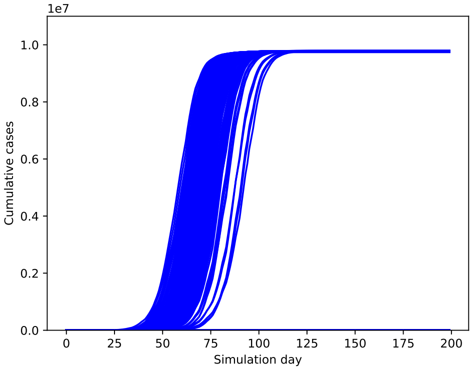

(4) $\alpha_c = 0.6$

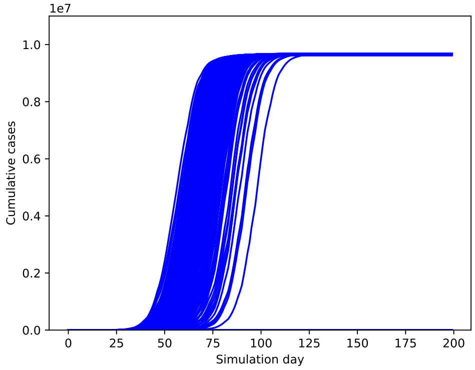

(5) $\alpha_c = 0.4$

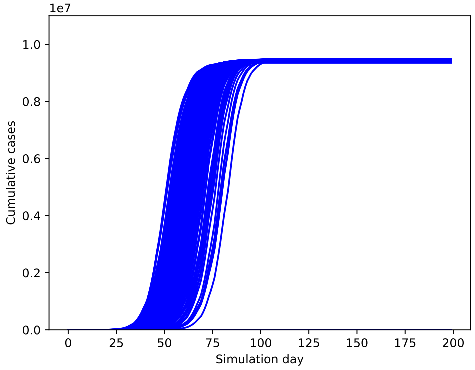

(6) $\alpha_c = 0.2$

Fig E. Evolution of the number of cumulative cases per day for different values of α_c for the Gamma distribution considered for the individual contact factor, for the scenario without interventions.

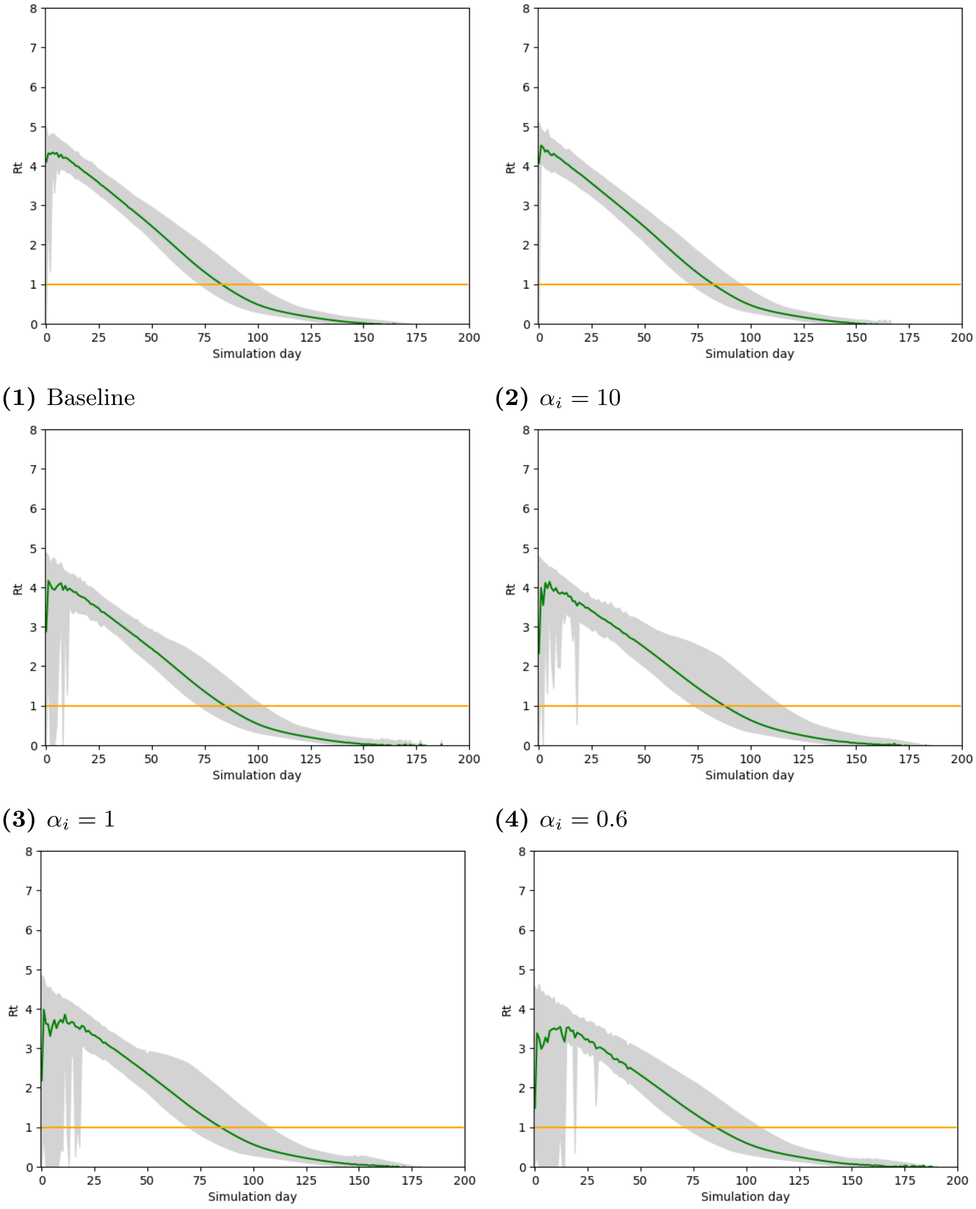

(1) Baseline **(2)** $\alpha_i = 10$

(3) $\alpha_i = 1$ **(4)** $\alpha_i = 0.6$

(5) $\alpha_i = 0.4$ **(6)** $\alpha_i = 0.2$

Fig F. Smoothed effective R_t per day when varying heterogeneity in infectiousness, for the scenario without interventions. The green line indicates the mean R_t per day, while the gray area represents the interval in which 95% of observations lie.

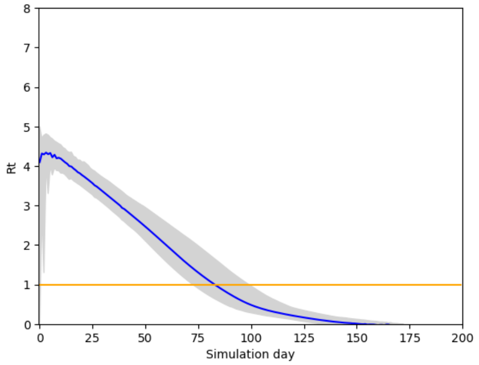

(1) Baseline

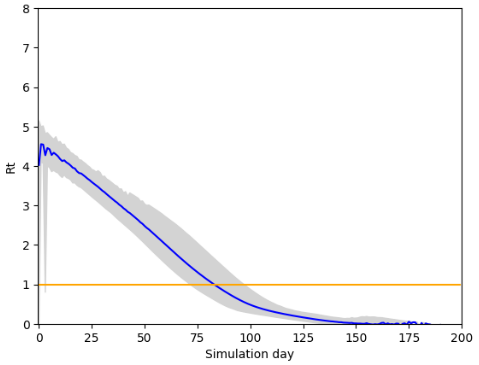

(2) $\alpha_c = 10$

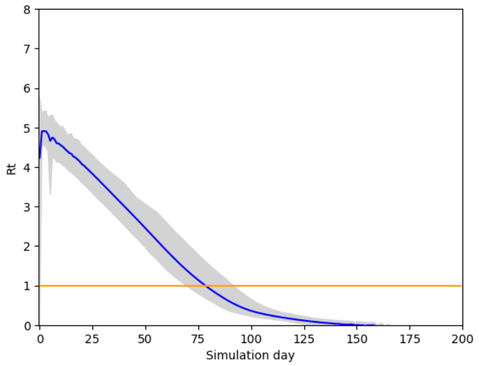

(3) $\alpha_c = 1$

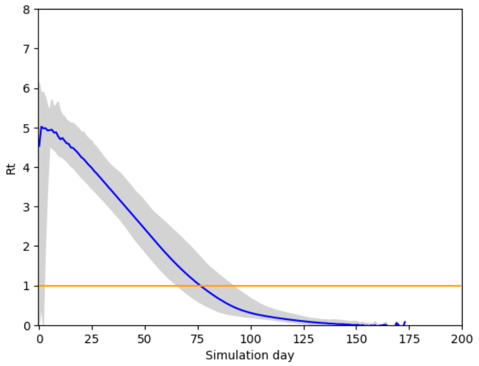

(4) $\alpha_c = 0.6$

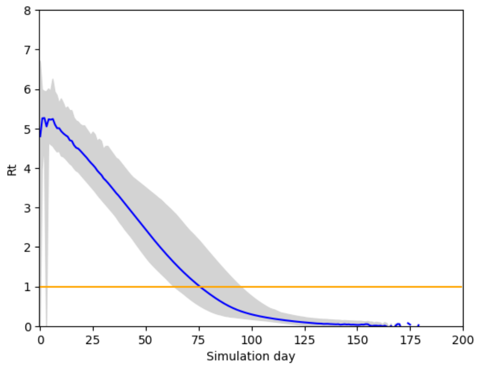

(5) $\alpha_c = 0.4$

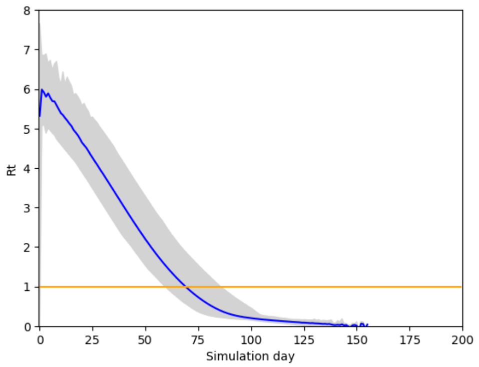

(6) $\alpha_c = 0.2$

Fig G. Smoothed effective R_t per day when varying heterogeneity in contact behavior, for the scenario without interventions. The blue line indicates the mean R_t per day, while the gray area represents the interval in which 95% of observations lie.

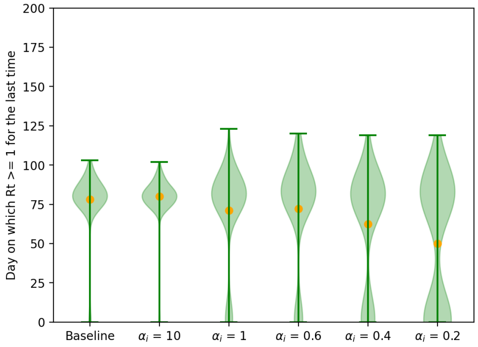

(1) Varying α_i for the Truncated Gamma distribution considered for the individual transmission probability.

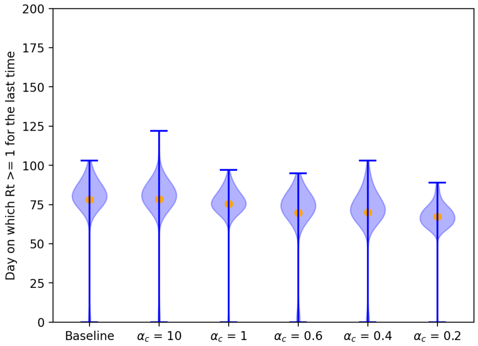

(2) Varying α_c for the Gamma distribution considered for the individual contact factor.

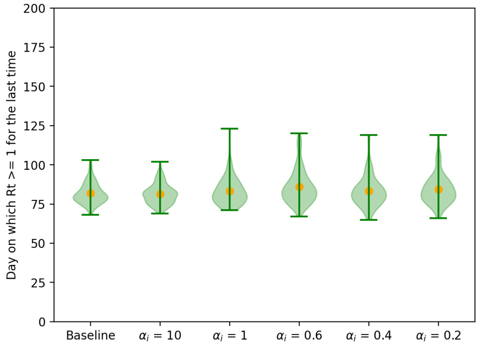

(3) Varying α_i for the Truncated Gamma distribution considered for the individual transmission probability. Runs with led to extinction (< 20 cases) were excluded.

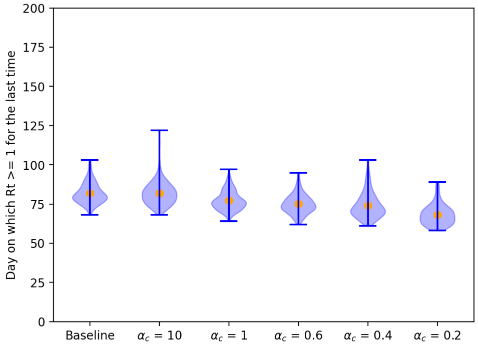

(4) Varying α_c for the Gamma distribution considered for the individual contact factor. Runs with led to extinction (< 20 cases) were excluded.

Fig H. Violin plots for the day on which the herd immunity threshold is reached for the different scenarios without interventions, over all simulations runs (panel 1–2) and only for simulations runs that generate more than 20 cases (panel 3–4). Scenarios in which α_i is varied are displayed in green, while scenarios in which α_c is varied are displayed in blue. Orange dots represent the means of the simulated values.

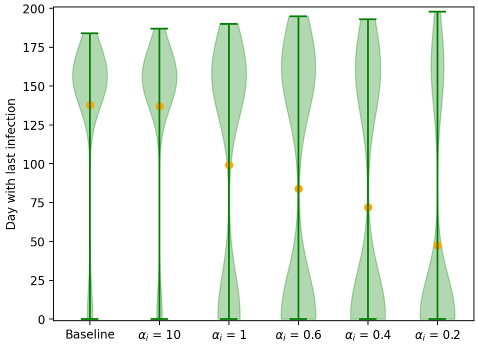

(1) Varying α_i for the Truncated Gamma distribution considered for the individual transmission probability.

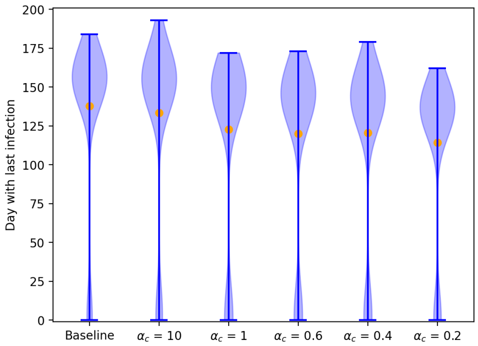

(2) Varying α_c for the Gamma distribution considered for the individual contact factor.

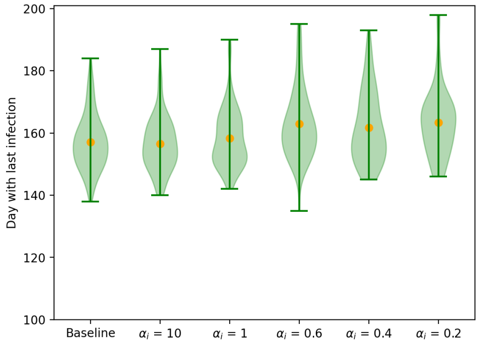

(3) Varying α_i for the Truncated Gamma distribution considered for the individual transmission probability. Runs with led to extinction ($<$ 20 cases) were excluded.

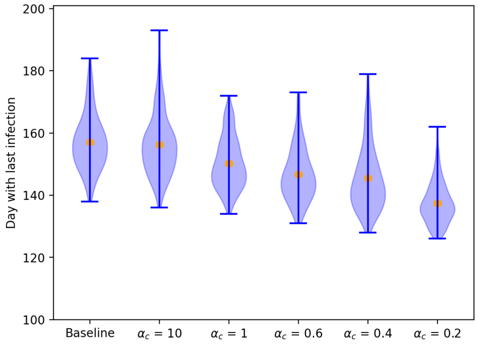

(4) Varying α_c for the Gamma distribution considered for the individual contact factor. Runs with led to extinction ($<$ 20 cases) were excluded.

Fig I. Violin plots for the day on which the last transmission event is observed for the different scenarios without interventions, over all simulations runs (panel 1–2) and only for simulations runs that generate more than 20 cases (panel 3–4). Scenarios in which α_i is varied are displayed in green, while scenarios in which α_c is varied are displayed in blue. Orange dots represent the means of the simulated values.

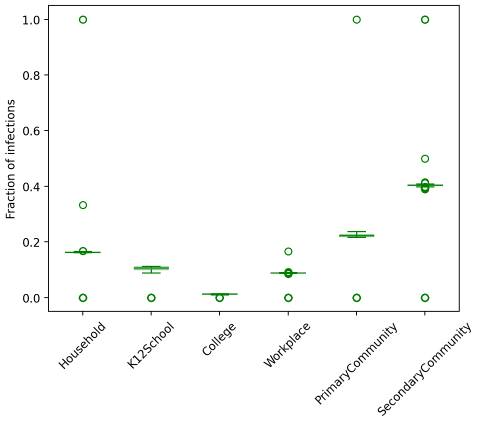

(1) Baseline

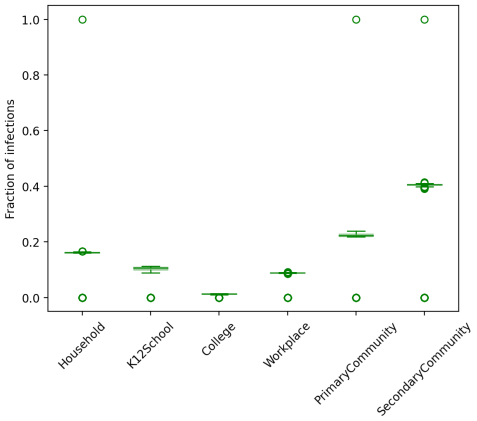

(2) $\alpha_i = 10$

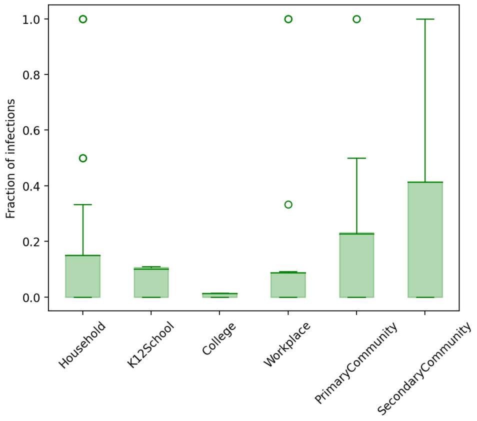

(3) $\alpha_i = 1$

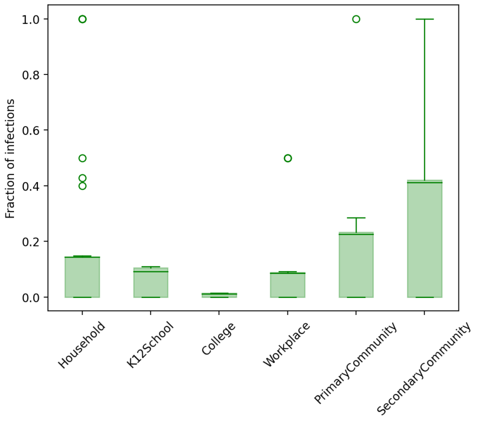

(4) $\alpha_i = 0.6$

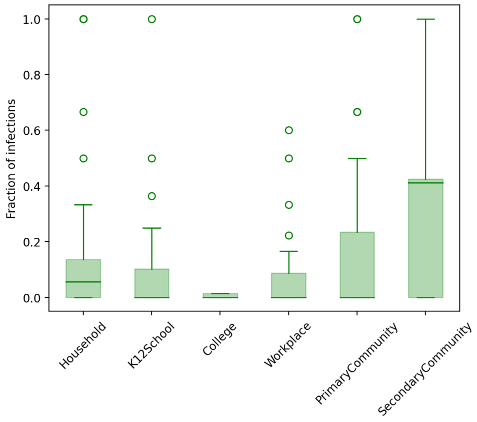

(5) $\alpha_i = 0.4$

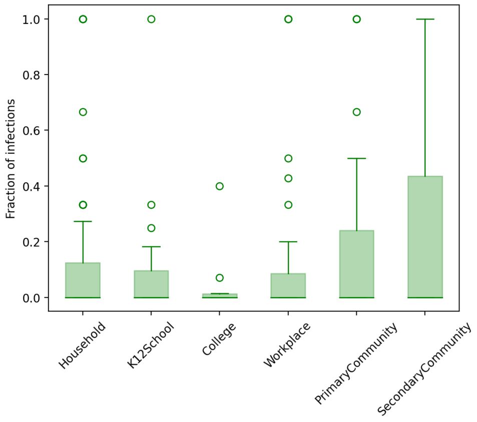

(6) $\alpha_i = 0.2$

Fig J. Proportion of transmissions per location type for different values of α_i for the Truncated Gamma distribution considered for the individual transmission probability, for the scenario without interventions.

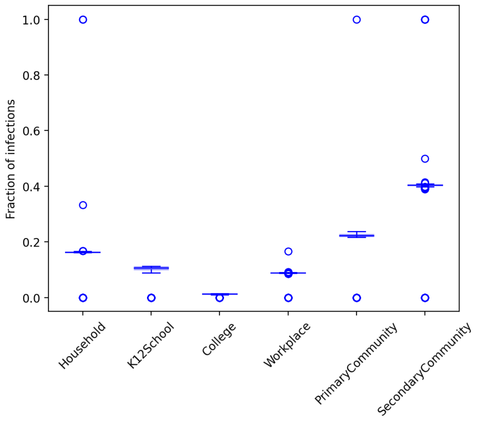

(1) Baseline

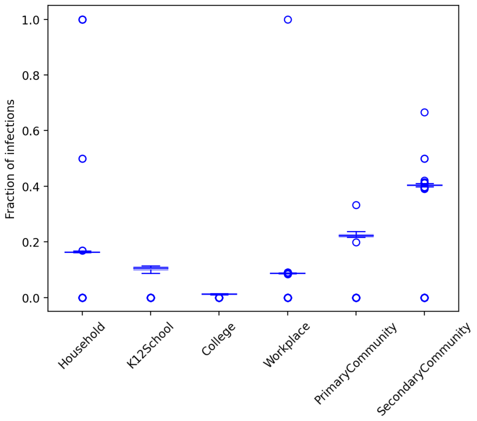

(2) $\alpha_c = 10$

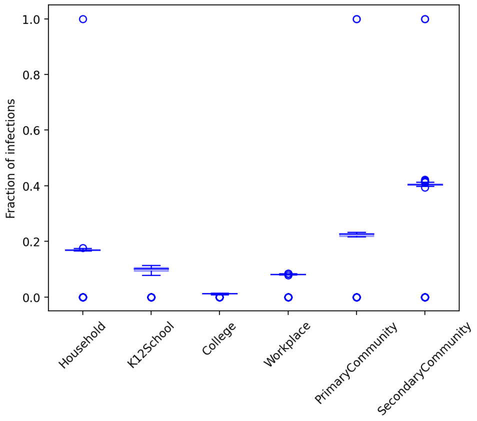

(3) $\alpha_c = 1$

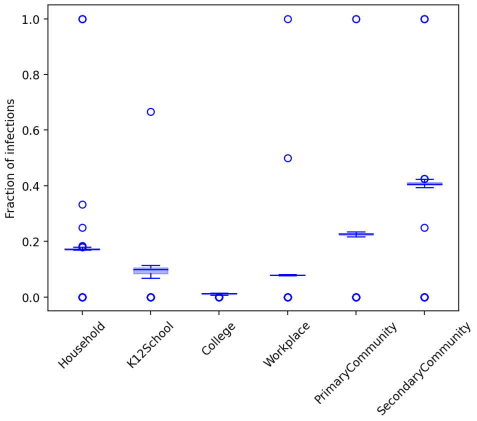

(4) $\alpha_c = 0.6$

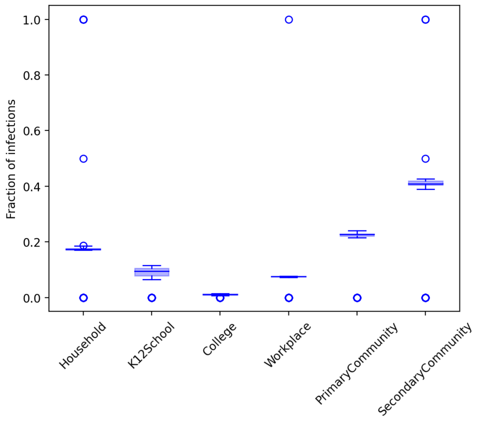

(5) $\alpha_c = 0.4$

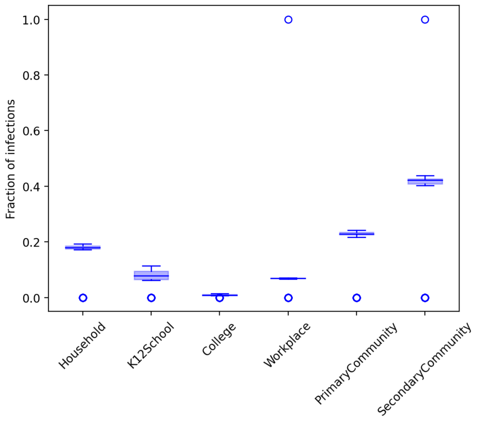

(6) $\alpha_c = 0.2$

Fig K. Proportion of transmissions per location type for different values of α_c for the Gamma distribution considered for the individual contact factor, for the scenario without interventions.

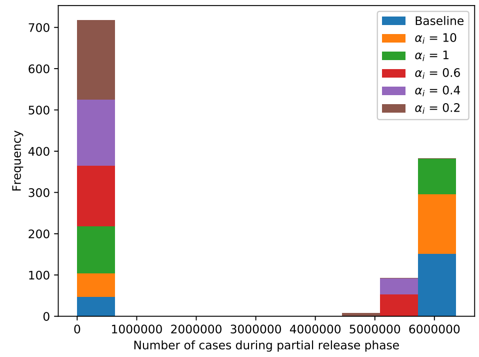

(1) Varying α_i for the Truncated Gamma distribution considered for the individual transmission probability.

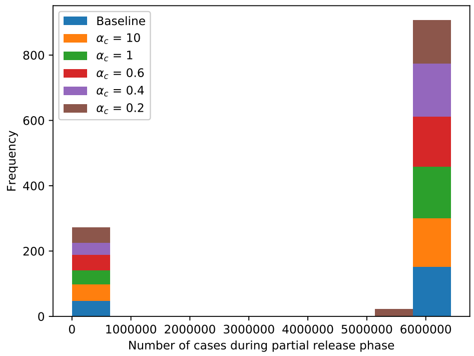

(2) Varying α_c for the Gamma distribution considered for the individual contact factor.

Fig L. Histograms of the number of cases during the partial release phase for the different scenarios regarding infectiousness-related and contact-related heterogeneity, for the scenario with social distancing.

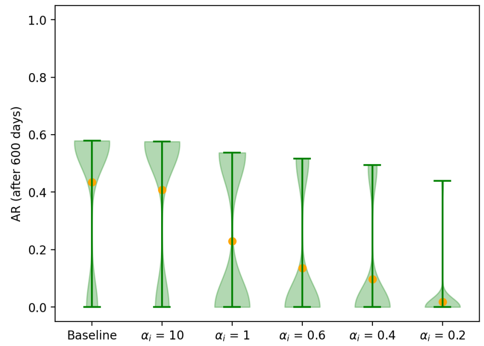

(1) Varying α_i for the Truncated Gamma distribution considered for the individual transmission probability.

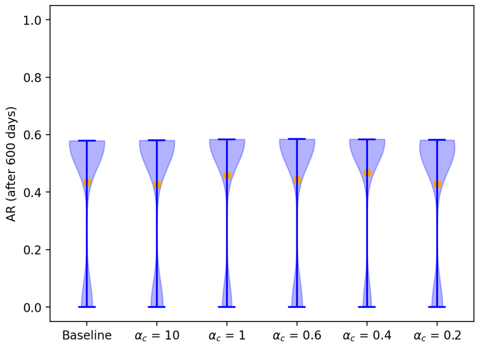

(2) Varying α_c for the Gamma distribution considered for the individual contact factor.

Fig M. Violin plots for the attack rate over 600 days for scenarios investigating the infectiousness-related heterogeneity (in green, panel 1) **and contact-related heterogeneity** (in blue, panel 2), **with social distancing**. The orange dots represent the mean attack rate across the simulation runs without extinction, i.e., simulation runs in which extinction occurs (< 20 cases) were excluded.

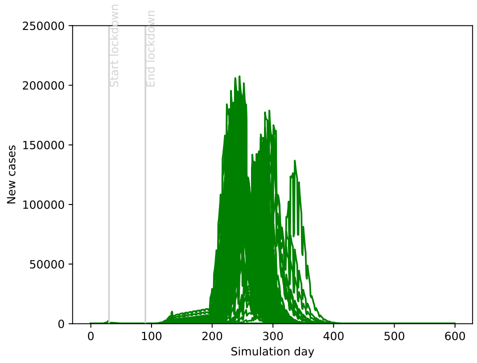

(1) Baseline

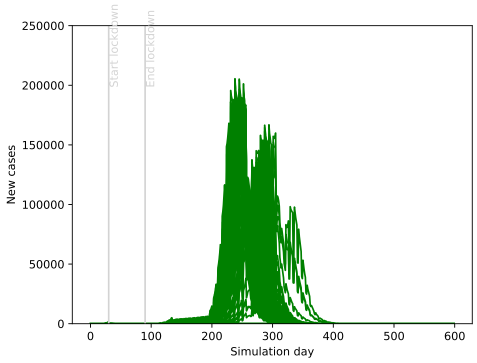

(2) $\alpha_i = 10$

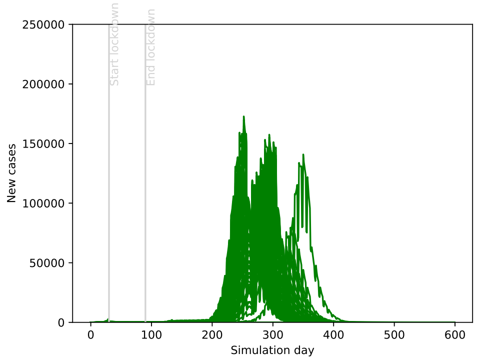

(3) $\alpha_i = 1$

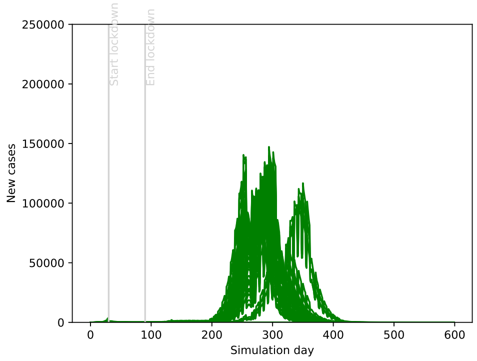

(4) $\alpha_i = 0.6$

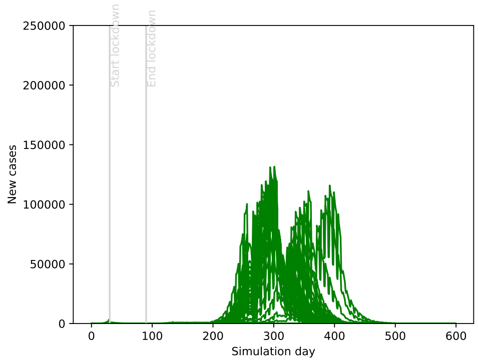

(5) $\alpha_i = 0.4$

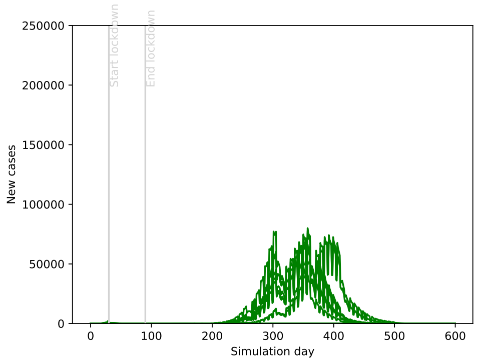

(6) $\alpha_i = 0.2$

Fig N. Evolution of the number of new cases per day for different values of α_i for the Truncated Gamma distribution considered for the individual transmission probability, **for the scenario with social distancing**.

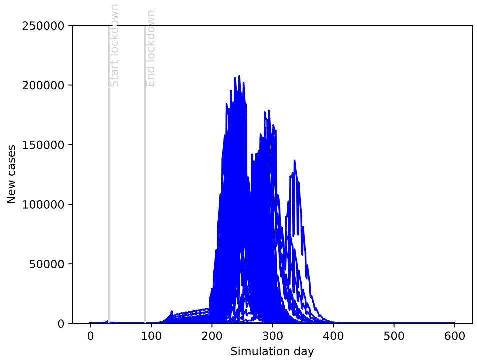

(1) Baseline

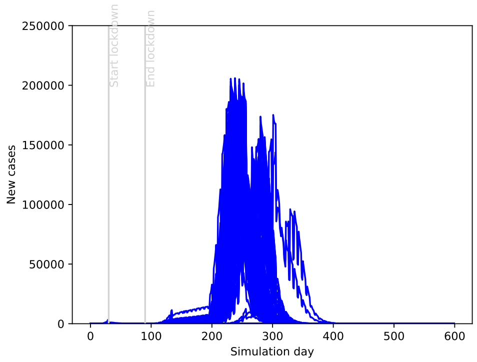

(2) $\alpha_c = 10$

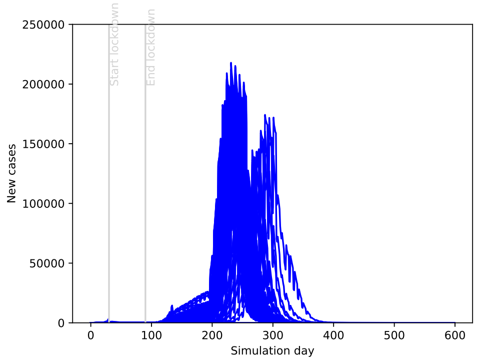

(3) $\alpha_c = 1$

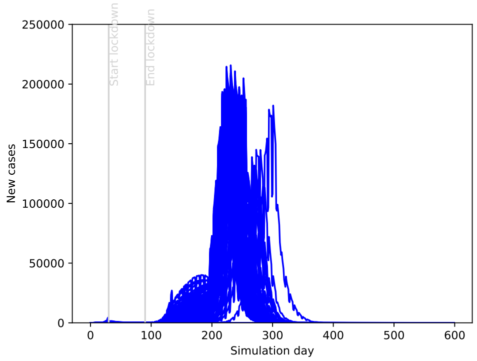

(4) $\alpha_c = 0.6$

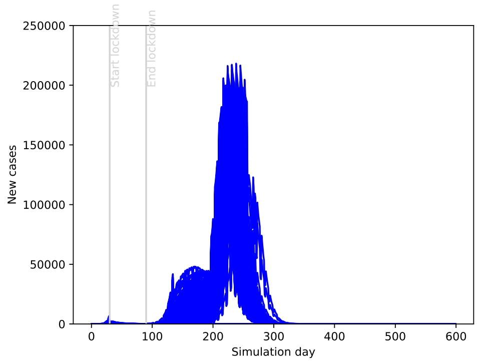

(5) $\alpha_c = 0.4$

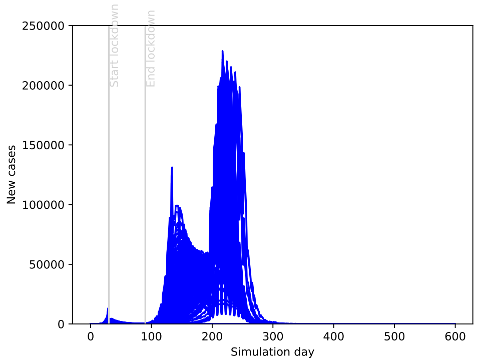

(6) $\alpha_c = 0.2$

Fig O. Evolution of the number of new cases per day for different values of α_c for the Gamma distribution considered for the individual contact factor, **for the scenario with social distancing**.

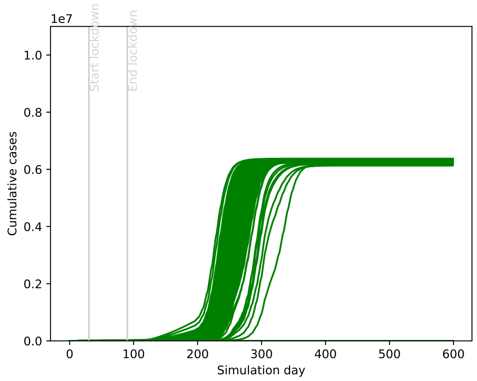

(1) Baseline

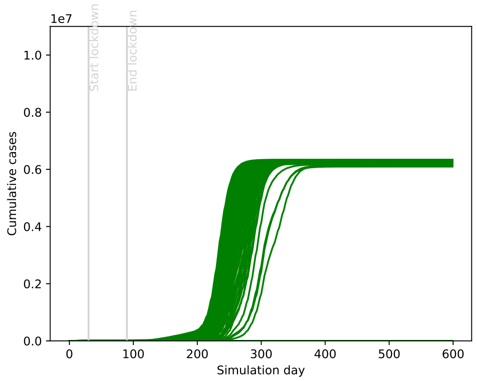

(2) $\alpha_i = 10$

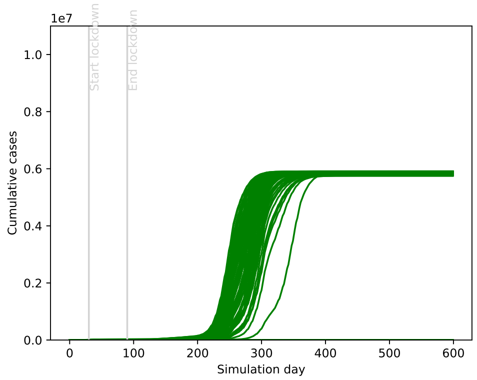

(3) $\alpha_i = 1$

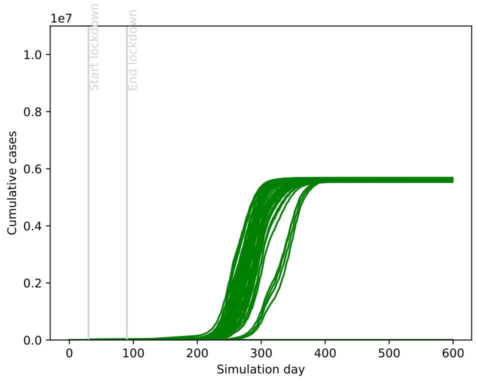

(4) $\alpha_i = 0.6$

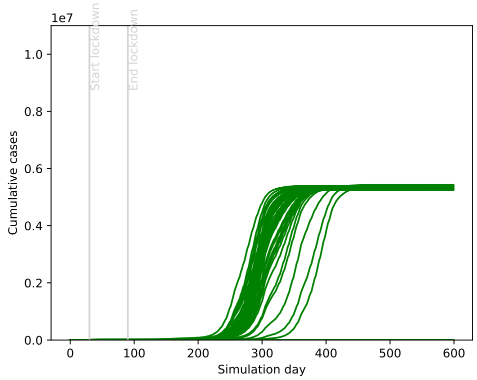

(5) $\alpha_i = 0.4$

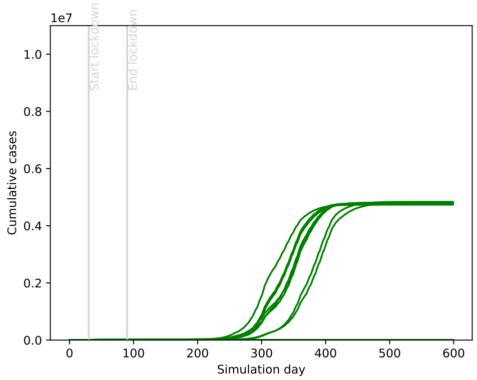

(6) $\alpha_i = 0.2$

Fig P. Evolution of the cumulative number of cases per day for different values of α_i for the Truncated Gamma distribution considered for the individual transmission probability, f**or the scenario with social distancing.**

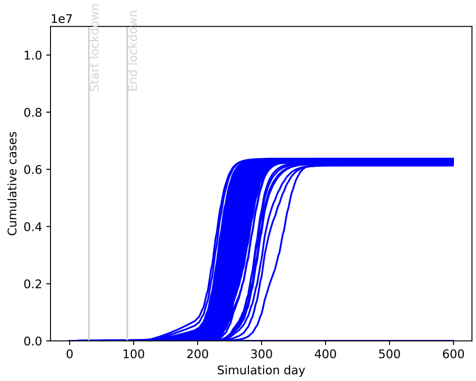

(1) Baseline

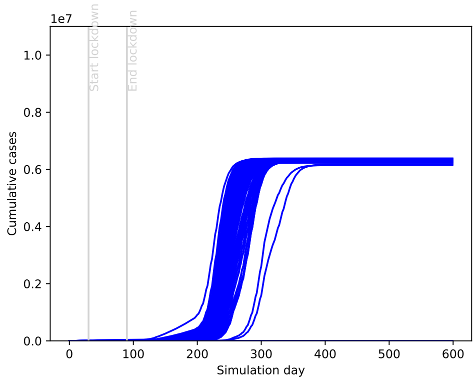

(2) $\alpha_c = 10$

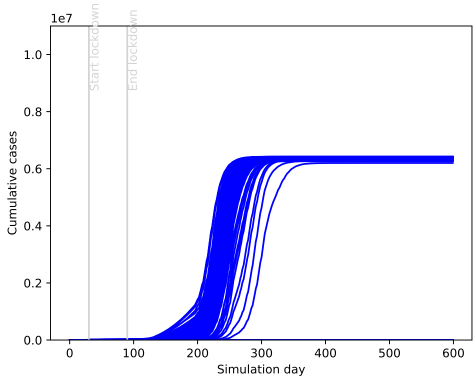

(3) $\alpha_c = 1$

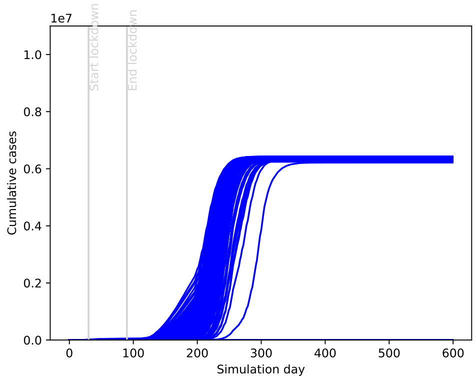

(4) $\alpha_c = 0.6$

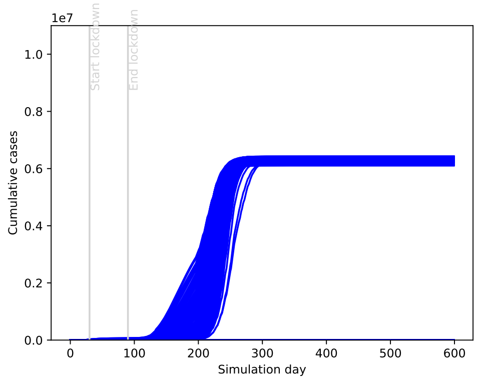

(5) $\alpha_c = 0.4$

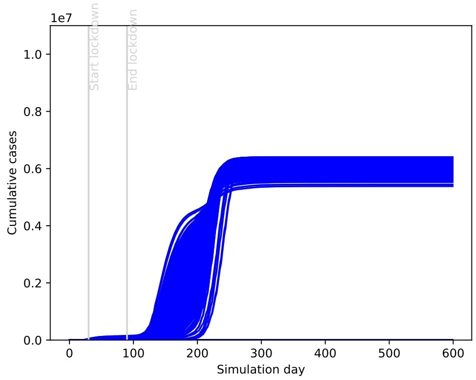

(6) $\alpha_c = 0.2$

Fig Q. Evolution of the cumulative number of cases per day for different values of α_c for the Gamma distribution considered for the individual contact factor, **for the scenario with social distancing.**

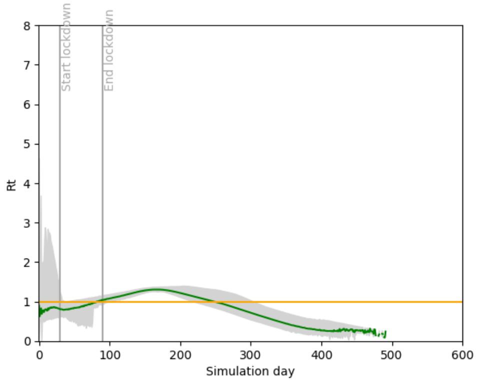

(1) Baseline

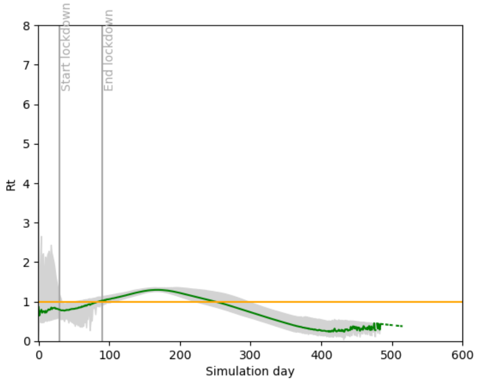

(2) $\alpha_i = 10$

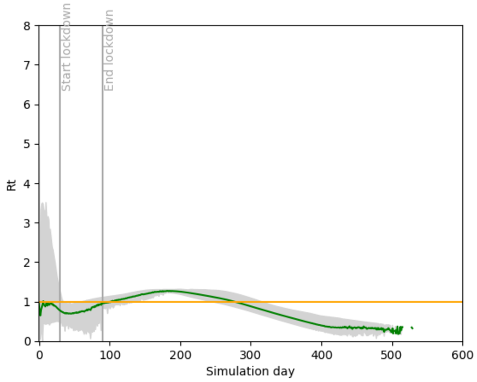

(3) $\alpha_i = 1$

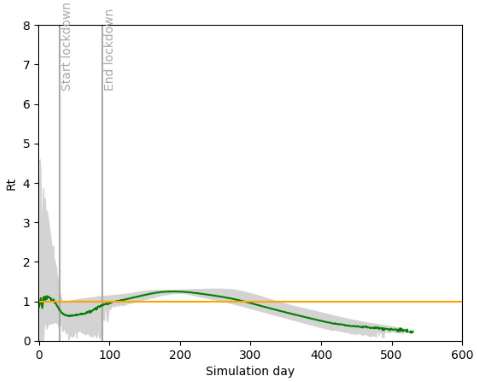

(4) $\alpha_i = 0.6$

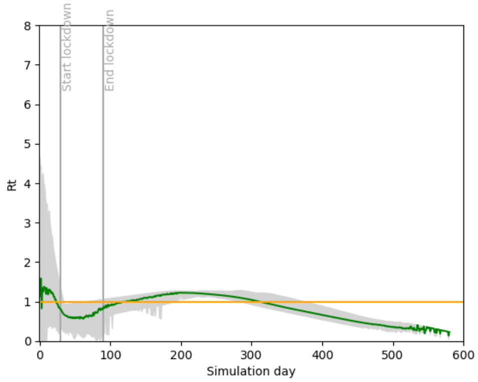

(5) $\alpha_i = 0.4$

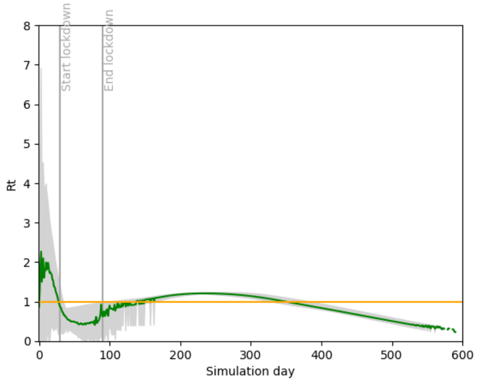

(6) $\alpha_i = 0.2$

Fig R. Smoothed effective R_t per day when varying heterogeneity in infectiousness, for the scenario with social distancing. The green line indicates the mean R_t per day, while the gray area represents the interval in which 95% of observations lie.

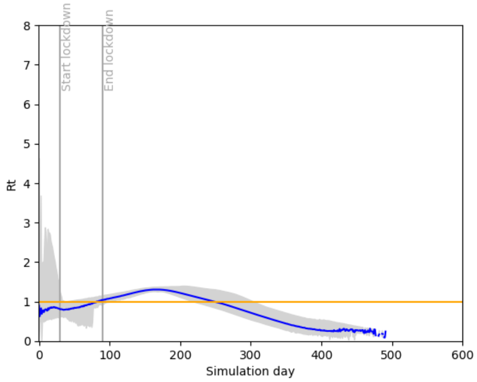

(1) Baseline

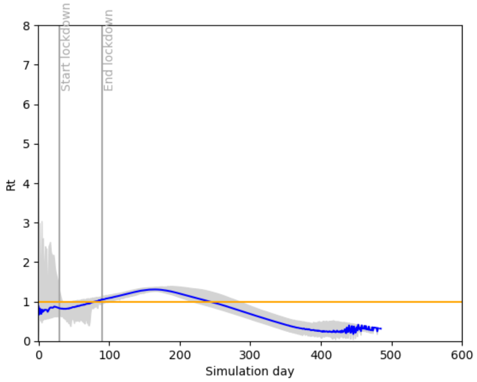

(2) $\alpha_c = 10$

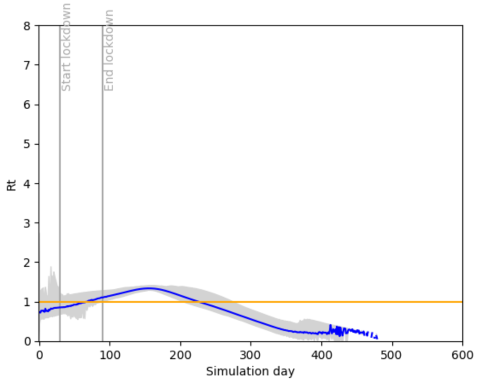

(3) $\alpha_c = 1$

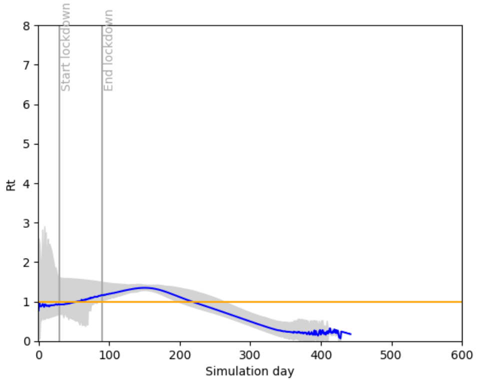

(4) $\alpha_c = 0.6$

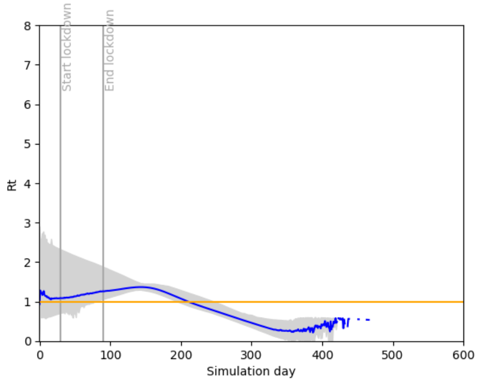

(5) $\alpha_c = 0.4$

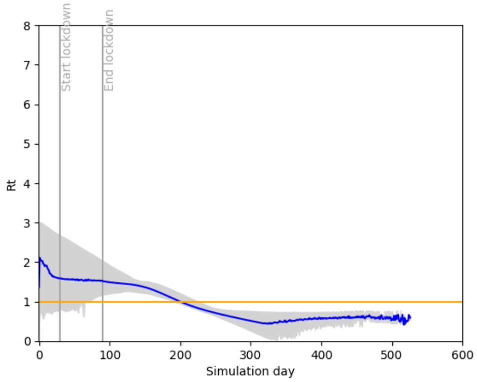

(6) $\alpha_c = 0.2$

Fig S. Smoothed effective R_t per day when varying heterogeneity in contact behavior, for the scenario with social distancing. The blue line indicates the mean R_t per day, while the gray area represents the interval in which 95% of observations lie.

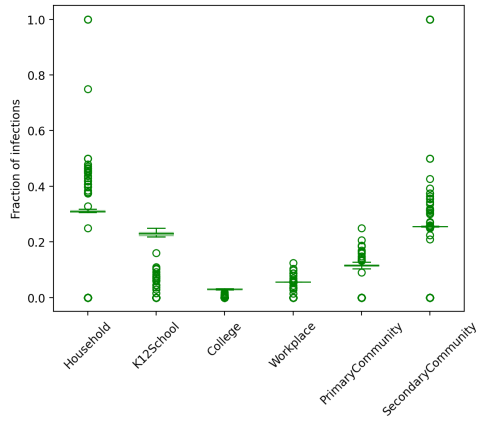

(1) Baseline

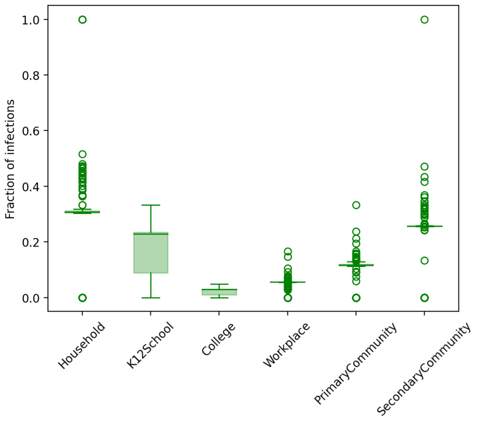

(2) $\alpha_i = 10$

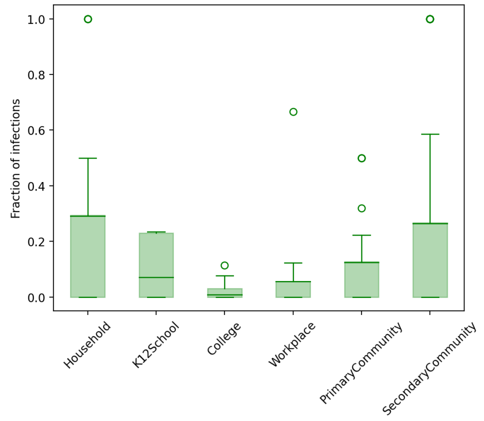

(3) $\alpha_i = 1$

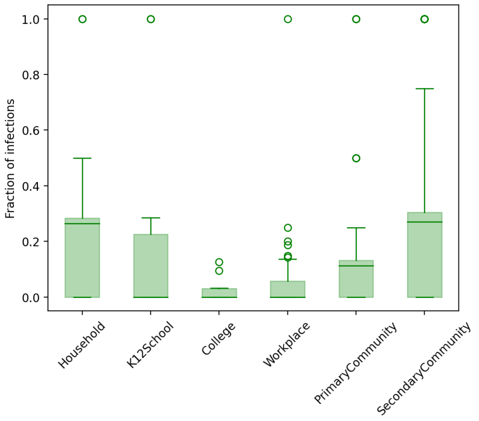

(4) $\alpha_i = 0.6$

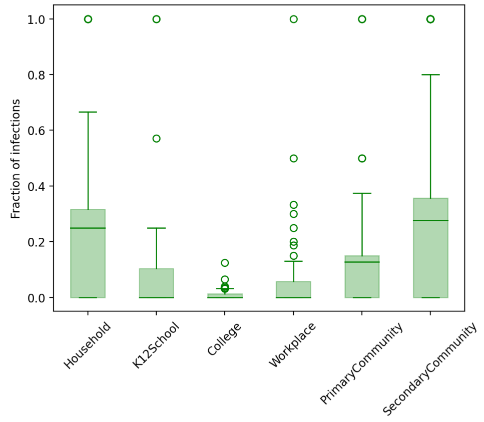

(5) $\alpha_i = 0.4$

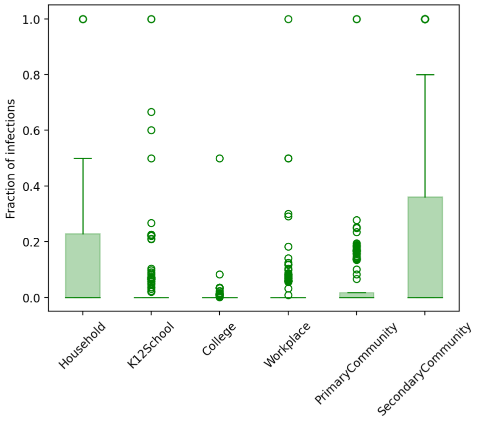

(6) $\alpha_i = 0.2$

Fig T. Proportion of transmissions per location type for different values of α_i for the Truncated Gamma distribution considered for the individual transmission probability, for the scenario with social distancing.

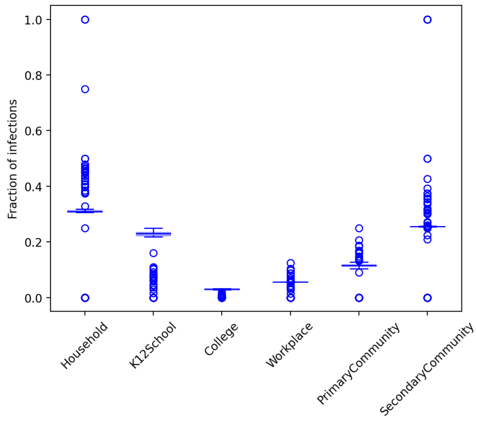

(1) Baseline

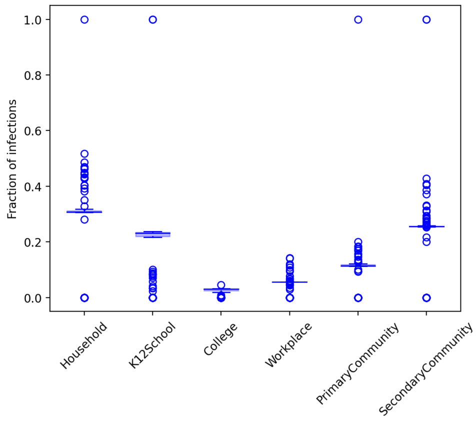

(2) $\alpha_c = 10$

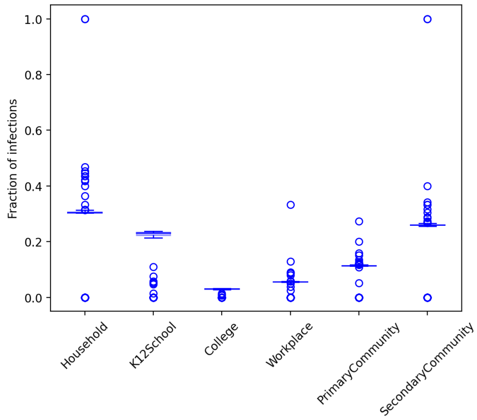

(3) $\alpha_c = 1$

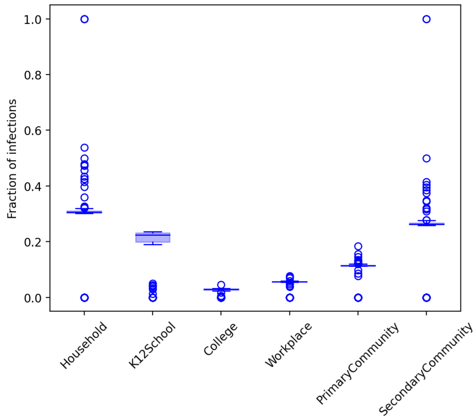

(4) $\alpha_c = 0.6$

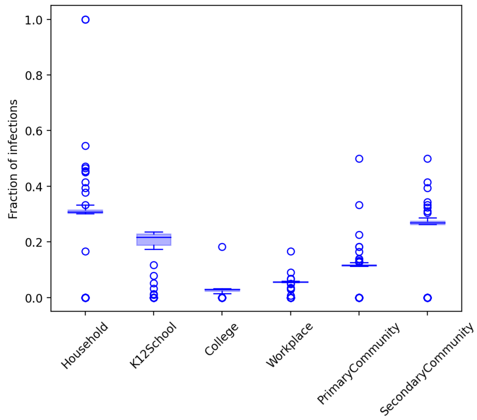

(5) $\alpha_c = 0.4$

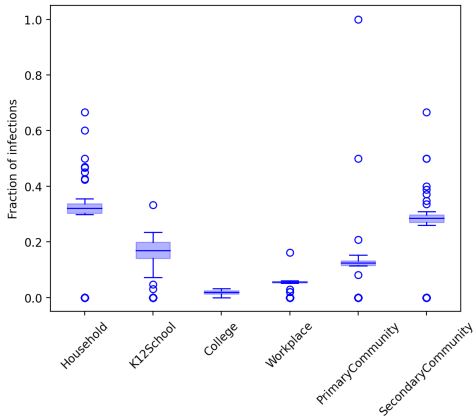

(6) $\alpha_c = 0.2$

Fig U. Proportion of transmissions per location type for different values of α_c for the Gamma distribution considered for the individual contact factor, for the scenario with social distancing.
