# Supplementary material for: Different forms of superspreading lead to different outcomes: Heterogeneity in infectiousness and contact behavior relevant for the case of SARS-CoV-2
Source: PLoS Comput Biol. 2022 Aug 22;18(8):e1009980. doi: 10.1371/journal.pcbi.1009980 (PMC9436127; doi:10.1371/journal.pcbi.1009980)
Supplement: Attachment — Submitted filename: reply_to_reviewers_20_06_2022.pdf [file pcbi.1009980.s006.pdf]

Dear Editor,

We are grateful for the feedback we received. We considered each comment and revised the manuscript accordingly. Please find here included a point-by-point reply to the reviewers' comments. We believe to have addressed all comments raised by the reviewers and hope you are willing to consider our revised manuscript for publication.

All changes in the manuscript have been marked in yellow. On behalf of all authors Yours sincerely,

Elise Kuylen

1 Reviewer 1

Thank you for the opportunity to review this manuscript. In this study the authors applied an agent-based model for SARS-CoV-2 transmission, with two distinct mechanisms for superspreading implemented in the model: heterogeneity in the infectiousness of individuals (implemented by drawing the infectiousness of an infected person from a Gamma distribution); and heterogeneity in contact rates (implemented by drawing a multiplicative contact parameter for each infectious person again from a Gamma distribution). The authors compared the impact of allowing for each type of superspreading event in two types of transmission scenarios (one with unconstrained social mixing, and one with restrictions gradually being lifted). They found that each of these types of superspreading produces different effects when considering the probability of importation generating an epidemic; the size of the resulting peak; the overall attack rate; timing of the peak; and the herd immunity threshold. I thought that the authors reflection was interesting that the reason contact-based heterogeneity causes a larger peak and reduced chance of extinction is due to more initial opportunities for the epidemic to be seeded and agree that this seems to be the case.

I found this to be a well-written study, that was clearly explained, with well-presented results. I think it is a useful contribution to the literature on models that account for superspreading, and some of those points identified in the limitations would be interesting to see explored in future work. I have only minor comments and requests for clarification.

Reply: We thank the reviewer for the positive appraisal of our work.

1. Abstract. "On the one hand, a higher level of infectiousness-related heterogeneity results in less outbreaks occurring following the introduction of one infected individual." Just to be very specific here – by fewer outbreaks, do you mean that the risk of an outbreak occurring following introduction of an infected individual into a susceptible population was lower? (From reading the methods I now see that this is the case but worth being clear

in the Abstract).

Reply: Thank you for your comment. Indeed, a higher level of infectiousness-related heterogeneity results in a lower risk of experiencing an outbreak that persists following the introduction of one infected individual into the population. We clarified this in the Abstract of the revised manuscript, and reformulated the sentence as follows: "On the one hand, a higher level of infectiousness-related heterogeneity results in a lower risk of an outbreak persisting following the introduction of one infected individual into the population.".

2. How did you confirm that the overall level of transmission within each of the two superspreading scenarios is the same, when you were adjusting the infectiousness parameter versus the contact rate parameter (i.e. to ensure that you are comparing like-with-like)?

   Reply: Thank you for your remark. We compared the average number of secondary cases per index case between the scenarios, to verify that R_0 indeed remained the same, and that the differences we observed between the scenarios were solely caused by varying heterogeneities in infectiousness and contacts. In Figure S1 of the Supplementary Material, we show that the average number of cases caused by an index case remains relatively stable over the different scenarios, although the variance does change (which we expected). We provided more details about this part of our analysis in the main text of the revised manuscript, and refer more clearly to the Supplementary Material for the results of this analysis (see line 136–141).

3. It took me a little while to identify in your paper what you assumed about acquisition and waning of immunity (as my immediate response to the results was to ponder the connection with immunity). I see in the first paragraph of your limitations section you do fully describe your assumptions but I think this should be made clearer earlier in your manuscript.

   Reply: Thank you for this comment, we addressed this concern by extending our overview of the workings of STRIDE under the heading 'Implementation' in our 'Methods' section. This now includes a description of our assumptions about the acquisition and waning of immunity (see line 86–87).

2 Reviewer 2

Kuylen et al. have used a preexisting COVID-19 simulation package to study 2 different "forms" of superspreading and determine the epidemiological consequences of the overdispersion in both factors in the population. My summary of the present results is: In a theoretical scenario with baseline contact related heterogeneity (CrH) but increased infectiousness-related heterogeneity (IrH), a simulation initiated by a single infected individual had a higher extinction probability, a delayed and decreased peak of infections, and a lower herd immunity threshold. In a parallel scenario with baseline IrH but increased CrH, peaks were earlier and higher, and herd immunity thresholds were higher. Resurgence after a lockdown increased with CrH but decreased with IrH.

The manuscript is very well written, and I found the results intuitive and the conclusions mostly justified by the results. I personally think the authors should do a bit more to take this work to a really high level – it is already good but just misses out on some exciting opportunities and I don't think should be too much extra work.

Reply: We thank the reviewer for these kind words about our work.

My major comments are:

1. Most importantly, the conclusions could be strengthened and made much more generalizable by using a global sensitivity analysis. I think the current version teaches us how each mechanism works independently, but I think you really miss out on the opportunity to comment on what happens if both change in a scenario. If you can run some grid beyond Table 1 and try to see if these effects can cancel out (ie are not identifiable if like IrH goes up but CrH goes down), or if they can amplify in any surprising ways, this would really bring it to the next level. I know the means are varied in the supplement, but I think the heterogeneities and means should all be simultaneously varied and a typical global approach applied (see Sobol/Morris or more simple LHS protocols for example).

   Reply: Thank you for this suggestion. We used an LHS design to test combinations of α_i and α_c. We tested a grid of 64 combinations, and added details about the methods and results in Supplementary Material S4. We found that the effects we observed when testing different values for α_i and α_c separately were largely conserved when we tested combinations of α_i and α_c. The extinction probability is still dominated by α_i (increasing for lower values of α_i), while there was no discernible effect of α_c. Although the attack rate decreased for both lower values of α_i and α_c, the impact of α_c on the attack rate was much larger. The effects of α_i and α_c on the size and timing of the peak, the herd immunity threshold and the day on which the last transmission event was observed remained opposite. How-

ever, α_i had a bigger impact on the size of the peak and the herd immunity threshold, while α_c dominated the timing of the peak and the day of the last transmission event. We also added a short overview of this analysis and its most important results to the main text of the revised manuscript.

2. I thought there were some nice results in the supplement including agreement between simulations and analytical calculations. I thought maybe this is worth noting in the main body so that others could use your expressions.

   Reply: Thank you for your kind remark. We therefore emphasized our work on the theoretical descriptions in the main text (see line 164).

3. The definitions of infectiousness-related and contact-related still kind of miss the point you make about contact situation. Perhaps you should frame this as per-contact transmission probability heterogeneity, and allow this to contain the notion of more indoor gatherings?

   Reply: Thank you for your comment. The difference between contact situations (e.g., indoors versus outdoors) is indeed an important source of heterogeneity for the spread of an airborne pathogen such as SARS-CoV-2. However, we chose not to explicitly add this factor in our current work as it is venue-based and not individual-based. The contact- and infectiousness-related heterogeneity we investigated are based on the individual and thus do not change according to the setting. Even so, heterogeneity regarding the contact environment is implicitly included in the contact heterogeneity which we implemented. Nevertheless, the environment in which contacts occur does constitute an important factor in the transmission of airborne pathogens such as SARS-CoV-2, and deserves to be investigated in more detail. As such, we discussed this important avenue for future research in our 'Introduction' (line 41–45) as well as in our 'Discussion' (line 463–479) section. We also reformulated our description in the Methods section to better reflect the difference between the contact pools for which we use contact rate data, and contact situations, which have an impact on the transmission probability which we did not explicitly model.

4. I know you are trying to avoid "disentangling" these two effects, but I really think this misses an opportunity to tell a more applicable message. To me you have data in here that already help disentangle, for example: you could speculate on what you think happened between Alpha and Beta vs Omicron waves based on case data. Did the virus get more heterogeneous

in terms of infection probability? or were people just tired of restrictions and more superspreading heterogeneity occurred? I think the case curves look pretty different, omicron was a lot sharper both up and down in a lot of places.

Reply: Thank you for this interesting suggestion. Even though it would indeed be very interesting to investigate the impact of different forms of superspreading on concrete SARS-CoV-2 waves and variants, it is difficult to speculate on this without taking into account other factors, which might have played a role as well. For example, the emergence of different variants of concern, in the presence of potential differences in contact-related and infectiousness-related heterogeneity, and the translation to incidence data should account for build-up of natural and vaccine-induced immunity as well. Consequently, such an exercise would entail the inclusion of all aspects related to disease transmission in the population under study, which is considered beyond the scope of the current paper. However, we adapted the Discussion section to better elucidate some further avenues of research concerning the influence of different forms of superspreading on different phases of the pandemic (see line 422-431).

5. The figures were kind of unusual with the a) and b) legends above the actual legends. I think it could be a lot clearer (different colors or something) that left panels are always varying IrH and right are always varying CrH too.

   Reply: Thank you for your comment. We used different colors for scenarios in which IrH and CrH were varied to make the Figures clearer.

6. I didn't write much on text because in general it was clear and excellent, however, the abstract could be much stronger, see my comments below:

   - "Superspreading events play an important role in the spread of SARS-CoV-2 and several other pathogens." Ok, but you don't really mention the other pathogens for the rest of the paper
   - "Hence, while the basic reproduction number of the original Wuhan SARS-CoV-2 is estimated to be about 3 for Belgium, there is substantial inter-individual variation in the number of secondary cases each infected individual causes." Ok, this 3 seems oddly specific given that you don't talk about the magnitude of variation
   - "Multiple factors contribute to the occurrence of superspreading events: heterogeneity in infectiousness and susceptibility, variations in con-

tact behavior, and the environment in which transmission takes place." Why bring up 3 if you only study 2?

- "While superspreading has been included in several infectious disease transmission models, our understanding of the effect that these different forms of superspreading have on the spread of pathogens and the effectiveness of control measures remains limited." What do you mean? Need to be more specific about what you think is missing, why do we need to know more and what precisely is not known?
- "To disentangle the effects of infectiousness-related heterogeneity on the one hand and contact-related heterogeneity on the other, we implemented both forms of superspreading in an individual-based model describing the transmission and spread of SARS-CoV-2 in the Belgian population." "the Belgian population" is not defined
- "We considered its impact on viral spread as well as on the effectiveness of social distancing." I'm not sure you really studied superspreading vs social distancing, more like resurgence probabilities after social distancing restrictions are lifted. If you wanted to talk about effectiveness of social distancing, you might want to study how often you see case loads of X level despite social distancing.
- "We found that the effects of superspreading driven by heterogeneity in infectiousness are very different from the effects of superspreading driven by heterogeneity in contact behavior." "very" is not specific enough, since you don't present absolute numbers (which I think you should), you should at least present percent increases or something
- "On the one hand, a higher level of infectiousness-related heterogeneity results in less outbreaks occurring following the introduction of one infected individual. Outbreaks were also slower, with a lower peak which occurred at a later point in time, and a lower herd immunity threshold. Finally, the risk of resurgence of an outbreak following a period of lockdown decreased. On the other hand, when contact-related heterogeneity was high, this also led to smaller final sizes, but caused outbreaks to be more explosive in regard to other aspects (such as higher peaks which occurred earlier, and a higher herd immunity threshold). Finally, the risk of resurgence of an outbreak following a period of lockdown increased."
  - fix less vs fewer
  - is "outbreaks" really right? I'm not an epi specialist, but I think this word is kind of dangerous
  - "also led to smaller final sizes" do you mean peaks? or is this prevalence? was prevalence lower after a simulation with increased IrH
- "Determining the contribution of both source of heterogeneity is therefore important but left to be explored further."

- Like I said above, this just doesn't feel right, I think you can do a bit of this here instead of saving for later.
- Whatever you decide, this should not be the last sentence in your abstract, end on something strong about the utility of your study (which is really nice!) rather than a lame concession of its limitations

Reply: Thank you for your constructive comments. We re-wrote the abstract in accordance with your suggestions.

3 Reviewer 3

I'm a theorist and I have never used STRIDE, so I'm trying to understand these results in terms of known results in network epidemiology. As recently discussed in a review https://arxiv.org/abs/2005.11283, it's important to distinguish forms of superspreading that affect the distribution of "out-degree" or "spread" - the number of secondary cases generated by a given infected individual - from those which affect to the "in-degree" or "risk" - the number of individuals from which a given individual can receive the infection. In some cases these are the same, since infections can spread in either direction along a social contact. But especially when different kinds of heterogeneity and interventions are taken into account, it's important to think about the network of contacts as partially directed. For instance, an individual with high viral load would have a high out-degree, while an immunocompromised and highly susceptible person would have high in-degree. As another example, masking is often claimed to reduce out-degree more than in-degree (i.e. protect others from the masked individual more than the other way around).

In particular, in networks where short loops are rare, the extinction probability is a function only of the out-degree distribution, while the epidemic size (and therefore the attack rate if it is measured over the entire epidemic) is a function only of the in-degree distribution. As a result, models of superspreading where individuals generate differing numbers of secondary cases, but where these secondary infections fall uniformly on all individuals in a given compartment, don't affect the epidemic size at all as long as R_0 is held constant. But they do affect the extinction probability, which increases as the variance in out-degree increases. Similarly, models where individuals have differing susceptibilities, but similar distributions of how many others they infect if they become infected, affect the epidemic size but not the extinction probability or epidemic threshold (again assuming R_0 is held constant).

So I'm trying to interpret Figures 2 and 3 in that context. Based on the analysis in https://arxiv.org/abs/2005.11283, it looks to me as if your infectiousness heterogeneity affects the "out-degree" or "spread", while your contact heterogeneity mainly affects the "in-degree" or "risk". This would explain why the extinction probability increases with infectiousness heterogeneity α_i but is essentially constant with respect to contact heterogeneity α_c. (In the text you

claim a slight increase and then a downward trend, but the error bars in Fig. 2b are consistent with no dependence on α_c at all.)

Similarly, in Fig. 3 the attack rate depends strongly on α_c, as we would expect if α_c affects the variance of the in-degree, but much less strongly on α_i. Is it possible that if you waited longer, so that you observed the entire epidemic curve — making the attack rate equal to the epidemic size — that this dependence would disappear completely?

On the other hand, in the Conclusion you say "However, when a person has a large number of contacts, they not only have more opportunity to infect others once they are infected, but they also have a disproportionally high chance to be exposed to infection themselves." So does your contact heterogeneity affect both risk and spread? I.e. does it increase the number of bidirectional contacts, along which the disease can spread in either direction? If so, this seems inconsistent with some of the experimental results.

As for the size and timing of the peak, I would argue that – if we condition on extinction not occurring – in the limit of large population size neither α_i nor α_c should make a difference, since once there are O(n) infected individuals the usual differential equation analysis should take over. In that case, the height and timing of the peak should depend only on R_0 and the matrix of mean contact rates between compartments, not on the distributions of contacts among individuals. Thus my guess is that the dependence in Fig. 4 is a finite-population effect.

That said, it is also true in branching process models that, if the variance in the degree distribution is large and we condition on the event that the process does not become extinct, then it has a higher average branching ratio at first (although this disappears after just a few generations if the underlying process is supercritical). This may explain your observation that the epidemic is more explosive at first — although I'm confused because I would expect this to depend more on α_i than α_c.

So to conclude: you have observed interesting distinctions between two types of superspreading in a popular epidemic simulation, and how they affect extinction probabilities, attack rates, and peaks in different ways. But since we know a lot about how epidemics depend on the structure of directed networks, it would be good to add a little more theoretical insight (which I think is available). That way the paper would be more convincingly about the effects of different mechanisms for superspreading, as opposed to just an observation about a particular simulation (i.e. STRIDE).

Reply: Thank you for pointing us to this interesting research. Even though STRIDE is not a network model, it does constitute networks and interesting parallels between our work and research on network models can indeed be drawn. The point that you make about the difference between 'in-degree' and 'out-degree' is an interesting one. It would be very useful to conduct a comparison between the results of the STRIDE model and results obtained through simulations with network models from the perspective of the distinction between in-

and out-degree. However, we believe that such an extensive comparison is beyond the scope of our paper. However, we did add the reference that you provided, as well as a short overview of important differences between our model and the models discussed in the review, to our Discussion section (see line 412–421).

In order to answer to your specific questions, we want to make the following clarifications. A very important distinction between the STRIDE model and the types of models that were discussed in Allard et al. (2020) is the fact that in STRIDE, the number of secondary cases that an infected individual can produce, is always limited by the contact pools they are a member of – regardless of the total population size. This has two important implications. First, when there are individuals in a given contact pool that have a higher level of infectiousness, other individuals belonging to the same contact pool also have a higher chance of becoming infected than individuals belonging to contact pools with no (or fewer) of such superspreaders. As such, this also alters the distribution of the 'in-degree', and this might explain why changes in infectiousness-related heterogeneity also have an impact on the final epidemic size - which in our results is indeed equal to the attack rate we present, as we run the simulations until the entire epidemic curve is observed. Secondly, the fact that the number of secondary cases per person is limited by the contact pools they belong to might also explain the fact that changes in infectiousness- and contact-related heterogeneity have an impact on the size and timing of the epidemic peak. It does seem to be the case that the 'out-degree' is more strongly affected by the infectiousness-related heterogeneity, hence the effect that is seen on the extinction probability (we also adapted our description of the effect of contact-related heterogeneity on the extinction probability, as indeed no clear effect is observable). We deem further parallels and differences between our model and the models discussed in the reference paper are very interesting to consider, but outside the scope of our current paper given that this would require quite a bit of additional work.

Minor comments:

- Using a right-truncated Gamma distribution for the individual transmission probability seems like a strange choice. Why not use, say, a Beta distribution, which also lets you choose both the mean and the variance while staying within the unit interval?

  Reply: Thank you for this comment. It is indeed true that different distributions could be used for our research (see, for example, Kremer et al., 2021). In future work, it would be beneficial to adapt distributions for contacts and individual infectiousness to available data. However, for the purpose of our – more methodological – research here, the nature of the dis-

tribution is of secondary importance, as long as it is possible to represent dispersion. We originally started using the Gamma distribution from the assumption that heterogeneity in the number of secondary cases per individual can be represented by a negative binomial distribution, or in other words, a Gamma mixture of Poisson distributions (which we originally assumed the contact distribution to be).

- "as the overdispersion parameter approaches infinity, the distribution becomes more centered around the mean." This phrase strikes me as strange, since as α increases the distribution has less dispersion, not more. Perhaps "shape parameter" is clearer.

  Reply: Thank you for this remark. You are right, we changed the way we formulated this in the main text of the revised manuscript: "as the shape parameter α approaches infinity, the dispersion of the distribution decreases, and the distribution becomes more centered around the mean".
